# Application of stem cells in tissue engineering for defense medicine

**DOI:** 10.1186/s40779-018-0154-9

**Published:** 2018-02-26

**Authors:** Chinedu Cletus Ude, Azizi Miskon, Ruszymah Bt Hj Idrus, Muhamad Bin Abu Bakar

**Affiliations:** 1 Bio-artifical Organ and Regenerative Medicine Unit, National Defence University of Malaysia, Sungai Besi Camp, 57000 Kuala Lumpur, Malaysia; 2 Department of Physiology, Pre-clinical Block, National University of Malaysia Medical Centre, Jalan Yaacob Latif, Bandar Tun Razak, 56000 Kuala Lumpur, Malaysia

**Keywords:** Tissue engineering, Defense medicine, Stem cells, War injuries

## Abstract

The dynamic nature of modern warfare, including threats and injuries faced by soldiers, necessitates the development of countermeasures that address a wide variety of injuries. Tissue engineering has emerged as a field with the potential to provide contemporary solutions. In this review, discussions focus on the applications of stem cells in tissue engineering to address health risks frequently faced by combatants at war. Human development depends intimately on stem cells, the mysterious precursor to every kind of cell in the body that, with proper instruction, can grow and differentiate into any new tissue or organ. Recent reports have suggested the greater therapeutic effects of the anti-inflammatory, trophic, paracrine and immune-modulatory functions associated with these cells, which induce them to restore normal healing and tissue regeneration by modulating immune reactions, regulating inflammation, and suppressing fibrosis. Therefore, the use of stem cells holds significant promise for the treatment of many battlefield injuries and their complications. These applications include the treatment of injuries to the skin, sensory organs, nervous system tissues, the musculoskeletal system, circulatory/pulmonary tissues and genitals/testicles and of acute radiation syndrome and the development of novel biosensors. The new research developments in these areas suggest that solutions are being developed to reduce critical consequences of wounds and exposures suffered in warfare. Current military applications of stem cell-based therapies are already saving the lives of soldiers who would have died in previous conflicts. Injuries that would have resulted in deaths previously now result in wounds today; similarly, today’s permanent wounds may be reduced to tomorrow’s bad memories with further advances in stem cell-based therapies.

**Electronic supplementary material:**

The online version of this article (10.1186/s40779-018-0154-9) contains supplementary material, which is available to authorized users.

## Background

Wounds resulting from injuries suffered during active military service represent major challenges to defense health systems, accounting for huge military expenditures in the US, Russia, Ukraine, Iraq, Syria, Afghanistan, Yemen, Sudan and other areas around the world with active conflicts [1–4]. The treatment options currently available include medication, surgical repair, transplants of allograft or xenograft tissue, artificial prostheses and mechanical devices. Unfortunately, most of these procedures can neither restore nor sustain a wounded tissue or organ over the long term; there is therefore an urgent need for lasting and complementary methods [5].

**Fig. 1:**
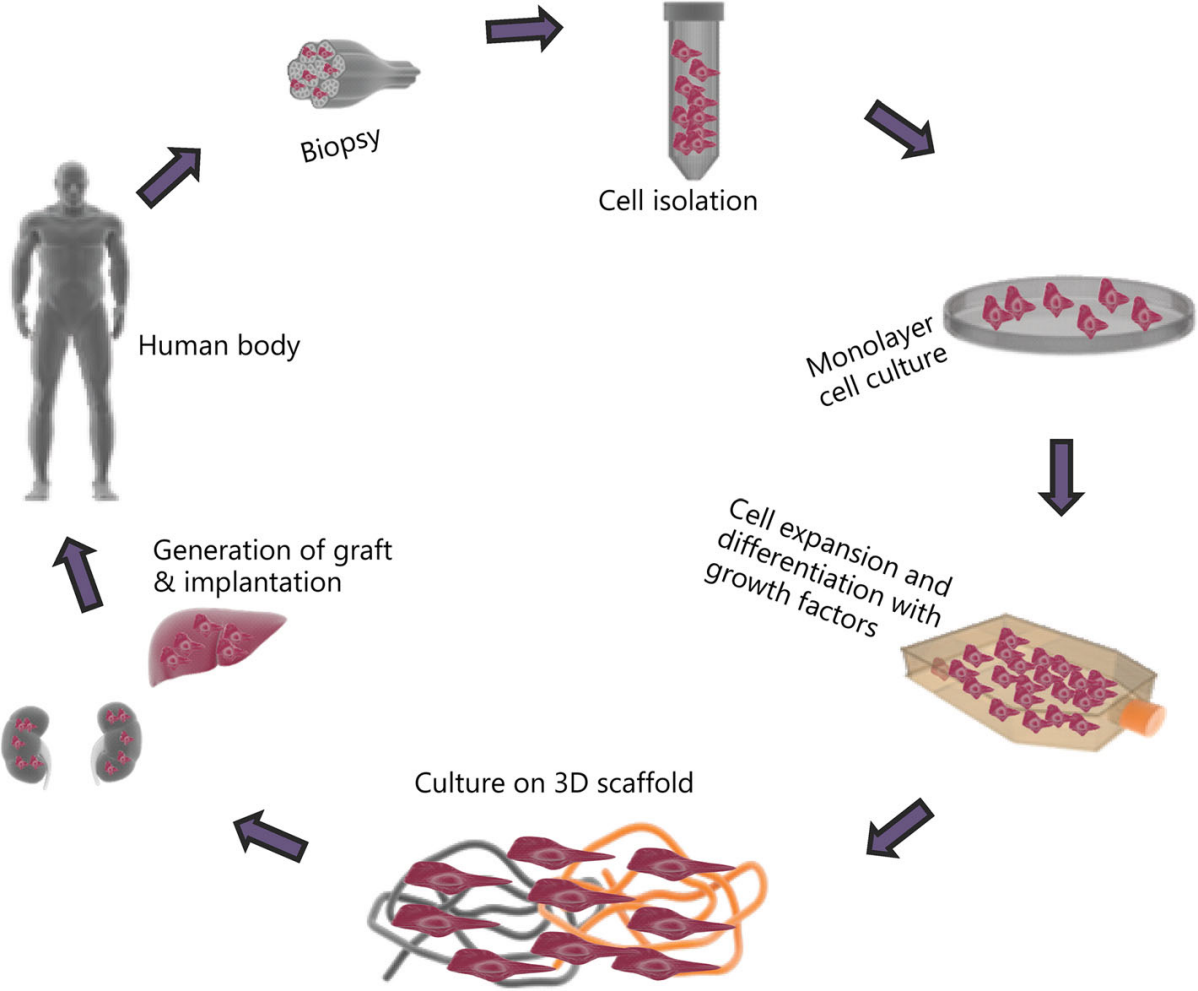
The basic materials and processes needed for tissue engineering technologies [6]

Tissue engineering is an emerging field representing potential alternatives to contemporary solutions. It is a science that combines stem cells, scaffolds with suitable growth factors, cytokines and chemokines to improve, replace or regenerate tissues and organs (Fig. 1) [6]. Through these techniques, organ failure can be addressed by the implantation of engineered, semi-synthetic tissues or whole organs that mimic the native function [7].

**Fig. 2:**
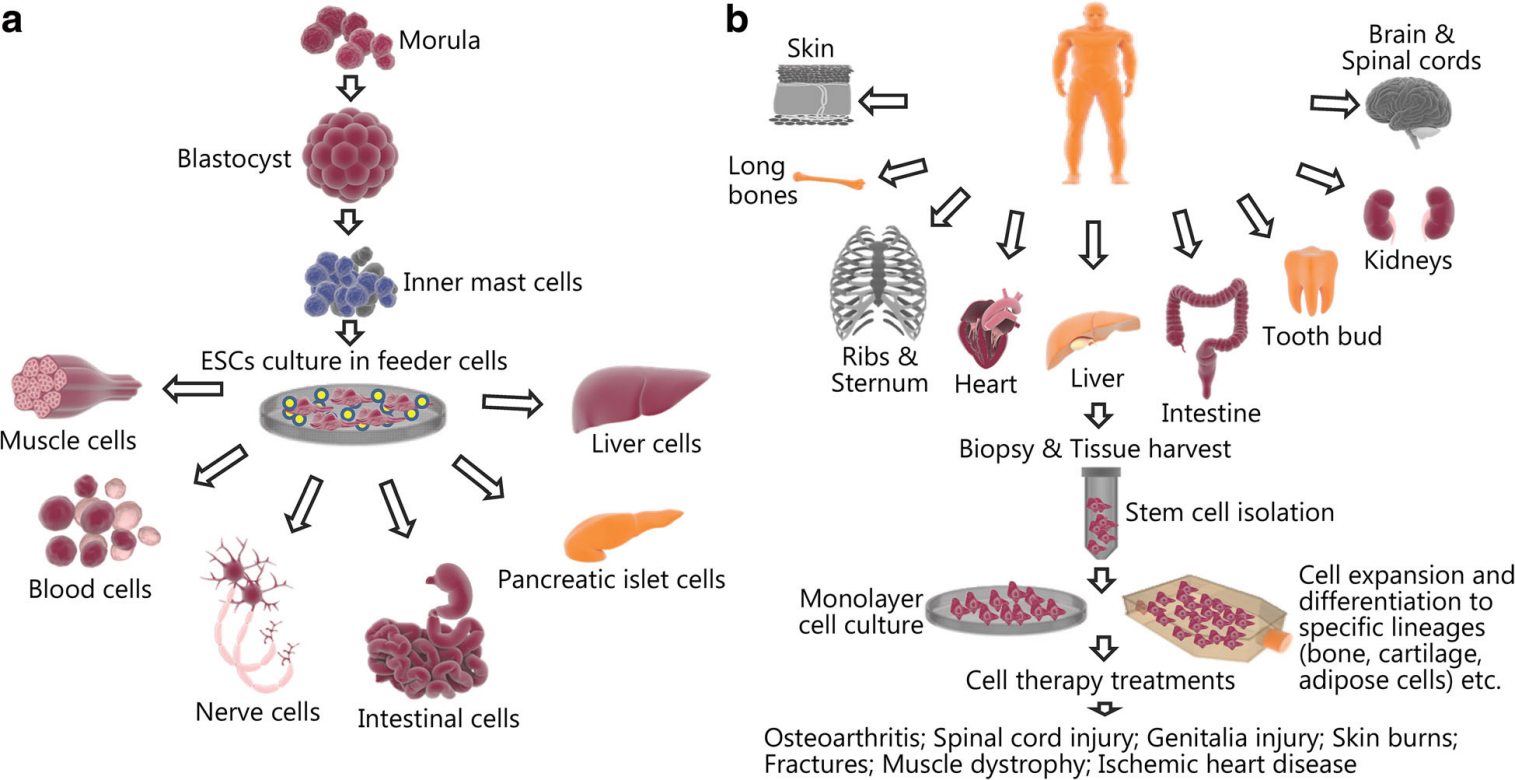
The source, harvesting procedure, culture and several potential uses of stem cells [8]. **a** Embryonic stem cells (ESCs); **b** Adult stem cells (mesenchymal stem cells, MSCs)

Stem cells (embryonic stem cells and adult stem cells) serve as the primary instrument of tissue engineering, a technology that has garnered a great deal of attention in civil and military research for providing possible treatment of many diseases and injuries (Fig. 2a) [8]. Mesenchymal stem cells (MSCs) are defined as adult native cells that have the ability to differentiate into tissues including, but not limited to, bone, cartilage and adipose cells in vitro (Fig. 2b). Studies have shown that their related anti-inflammatory, trophic, paracrine and immuno-modulatory functions tend to elicit even greater therapeutic effects in association with these cells. This in turn aid stem cells in restoring localized or systemic conditions for normal healing and tissue regeneration. In contrast to popular pharmaceutical agents that deliver a single specific dose at treatment sites, MSCs secrete and regulate bioactive factors and signals at variable concentrations in response to local micro-environmental cues [9].

Given the above functions, the inclusion of tissue engineering technology and stem cells in the treatment of battlefield injuries can bridge long-standing challenges in tissue repair and restoration. These possibilities provide promising therapeutic options to address the unmet needs of medical defense [10]. For instance, the complicated nature of present military capabilities, primarily the potential for nuclear warfare, necessitates the development of countermeasures that can address these challenges [10]. Since 1945, nearly every radiation injury has been caused by accidents in nuclear power plants and medical radiotherapy. Nevertheless, the proliferation of nuclear technology, the quest for procurement and enrichment of radioactive materials by additional countries, and the surge in terror groups, has intensified concerns regarding the possible use of nuclear weapon to inflict extensive civilian and military casualties [11]. Apart from the instant thermal destruction, the colossal damage of a nuclear strike would lead to acute radiation syndrome (ARS). This manifests as physical and chemical alterations to DNA, which affects the rapidly dividing cells of the hematopoietic system and gastrointestinal tract [12].

In this review, the treatments and potential applications of tissue engineering for injuries to the skin, sensory organs, nervous system tissues, the musculoskeletal system, circulatory/pulmonary tissues, and genitals/testicles, for acute radiation syndrome and for the development of biosensors are discussed. Search methods included references in the literature, cited sections of the most relevant articles, articles by authors of key references and science citation indexes, and other relevant web reports.

## Tissue/organ injuries

### Skin injuries

The skin, the largest organ of the body, possesses a complex multi-layered structure that guards the underlying muscles, ligaments, bones, and delicate organs [13]. It is made up of three layers: the epidermis, dermis, and hypodermis or subcutaneous tissue. It serves as the first line of defense against any external stimuli; it is therefore the most vulnerable and requires rapid regeneration.

Gunshot injuries, explosions, chemical exposures, nuclear heat, and any other agent of warfare can cause severe damage to the skin. Depending on the type and extent of an injury, the body regenerates itself through the process of wound healing, a dynamic process that involves stem cells, progenitor cells, parenchymal cells, extra cellular matrix (ECM), blood cells and soluble mediators in three phases: inflammation, proliferation and remodeling [14].

**Fig. 3:**
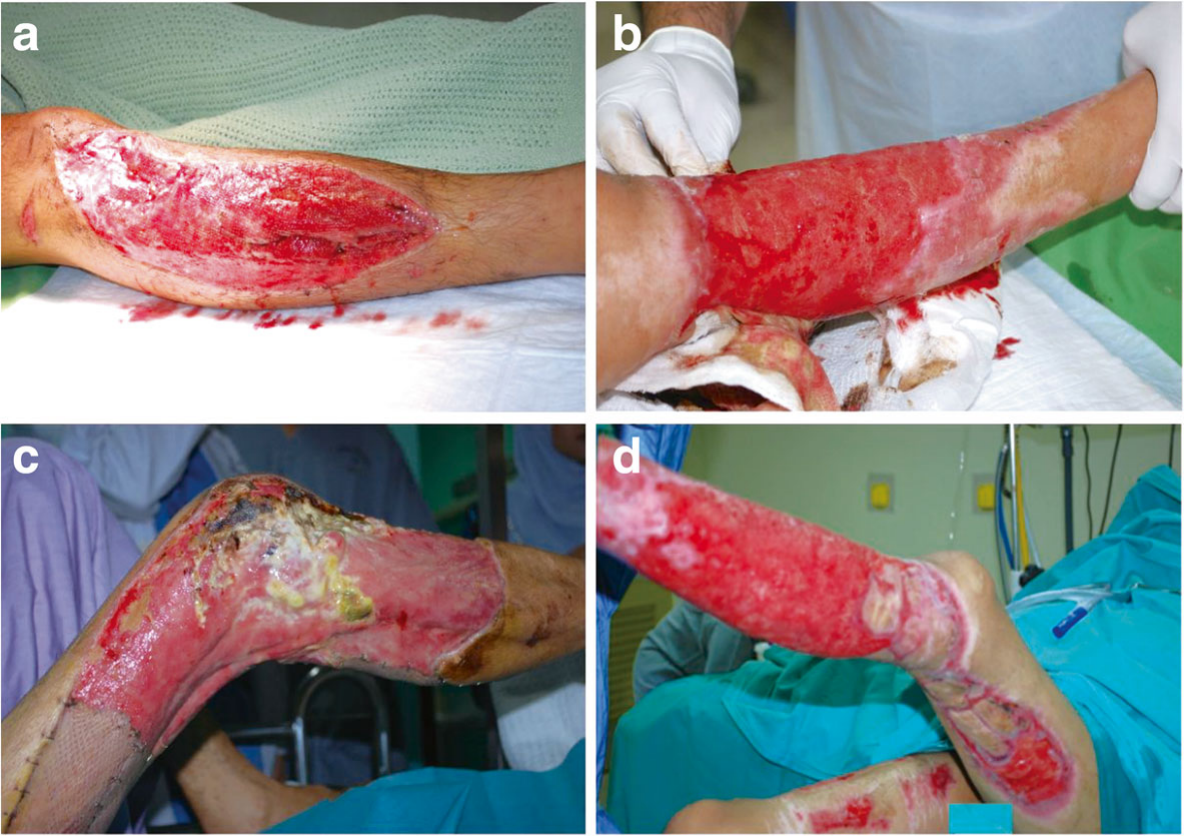
Major skin injuries including 3rd degree skin wounds where the whole skin has been lost that require a graft or tissue-engineered substitute (Courtesy of the Tissue Engineering Centre National University of Malaysia Medical Centre). **a** Severe wound from accident; **b** Wound sustained from burns; **c** Severe wound sustained from accident with necrosis; **d** Severe wound from burns with necrosis

The above processes can occur as long as any of the three layers of skin remain. Third- and fourth-degree burns are considered full thickness and are the most difficult to treat (Fig. 3). These require the regeneration of the entire skin structure. Apart from structural regeneration, restoring the function of the original skin is critical. Improved understanding of wound healing mechanisms has aided emerging tissue engineering technologies in the generation of functional skin [15, 16]. The current method of split skin graft (SSG), which costs over $50,000 to cover burn areas on up to 40% of an average man, suffers from several limitations, such as donor site morbidity, extended healing times and significant expense [13]. Efforts to overcome these limitations have led scientists to explore techniques for engineering skin substitutes.

Skin substitutes are defined as a heterogeneous group of substances that help in either temporary or permanent closure of any kind of wound. Skin substitutes help when standard surgical therapies are not desirable. The decision is case based, depending on the type and extent of burns, shallow or deep, and the area of the body affected [17].

**Fig. 4:**
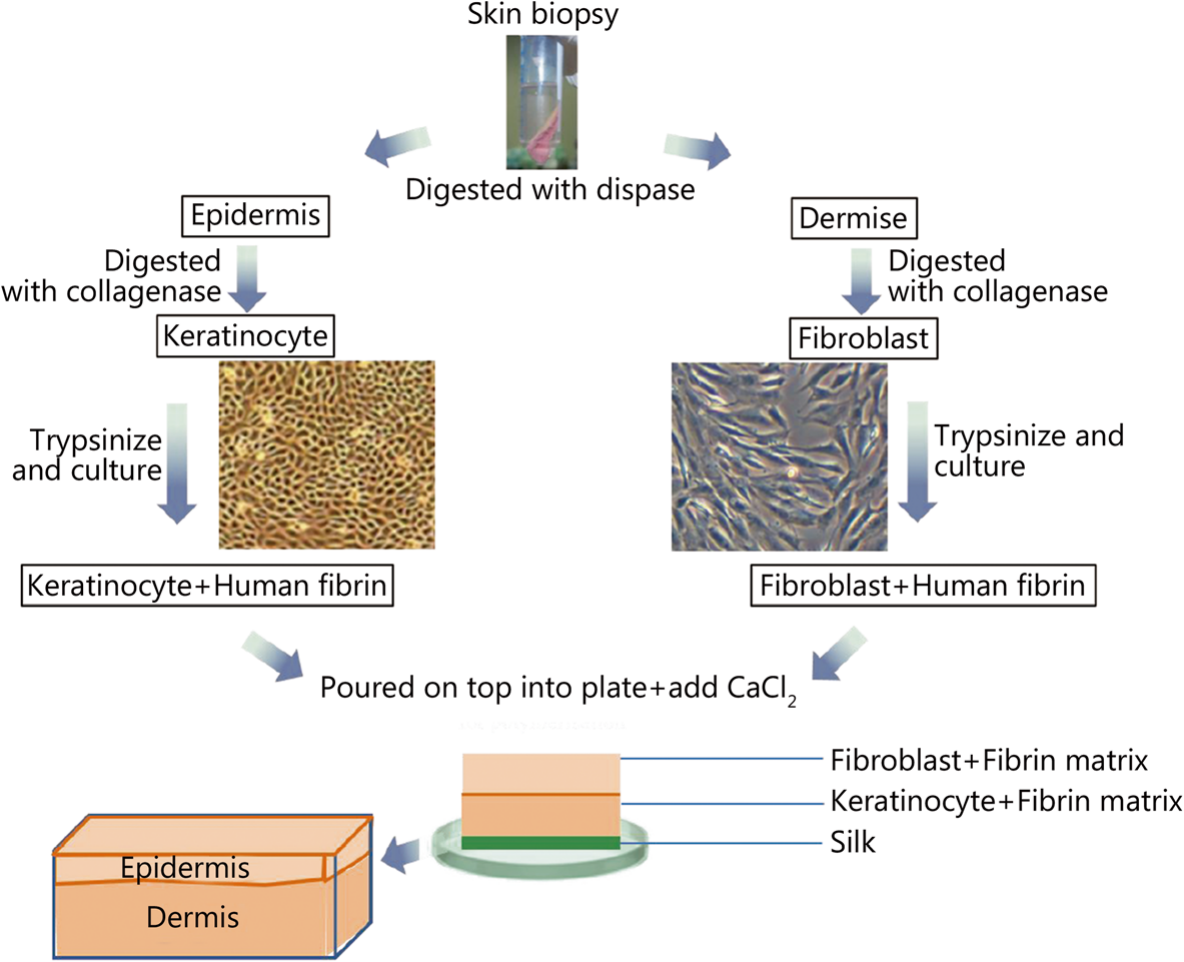
The major techniques of producing a bi-layered skin substitute. The major cells required are fibroblasts and keratinocytes, with fibrin functioning as a scaffold

A good skin substitute should possess the following qualities: is flexible in thickness, is durable for long-term wound stability, conforms to irregular wound surfaces, resists infection, prevents water loss or dehydration, withstands shear forces and wound hypoxia, is cost efficient and easy to prepare, store and use, and has wide availability and a long shelf life. It should be devoid of antigenicity, provide permanent wound coverage, and recreate dermal, as well as, epidermal components [13]. To date, there has not been a perfect substitute for skin created; however, the attempts that have been made have proved beneficial (Fig. 4).

**Fig. 5:**
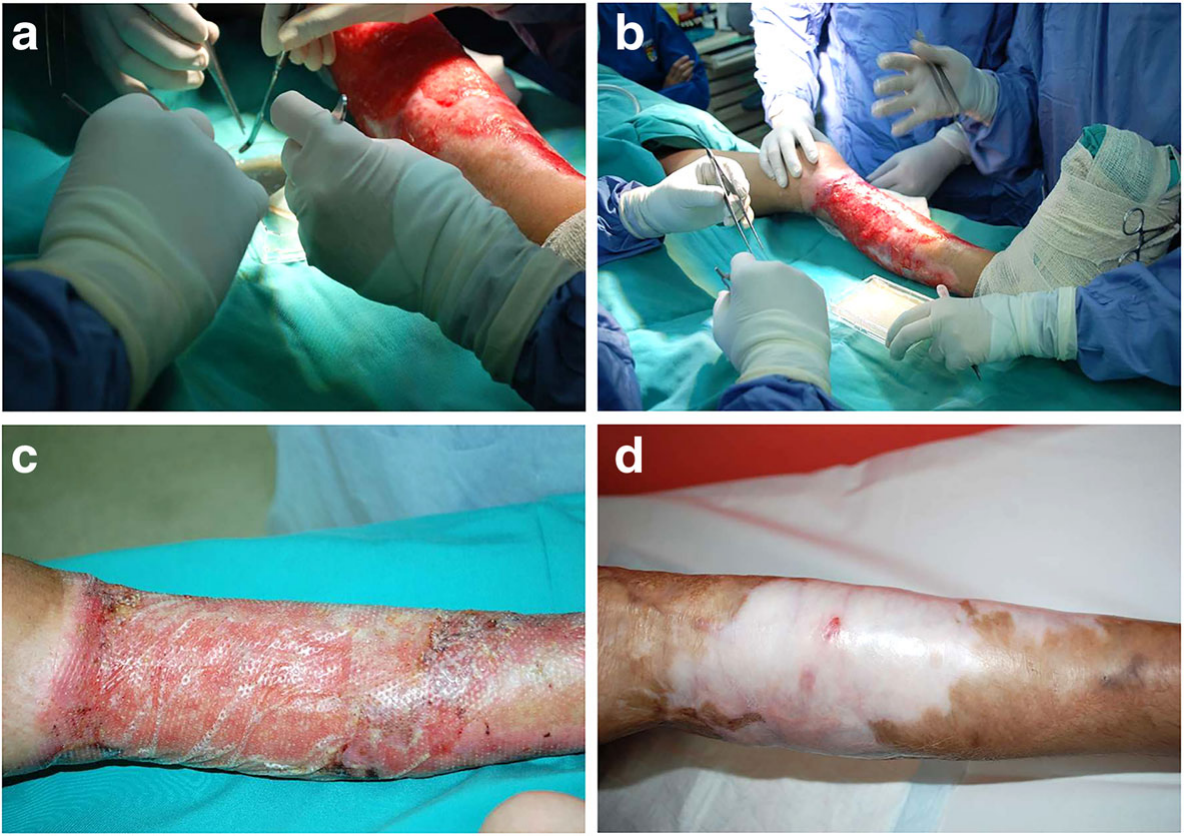
Treatment of a burn patient with a tissue-engineered bilayer skin substitute [24–26] (Courtesy of the Tissue Engineering Centre National University of Malaysia Medical Centre). **a** Medical team examining the skin substitute; **b** Fixation of the skin substitute; **c** One month post-treatment; **d** Three months post-treatment

**Fig. 6:**
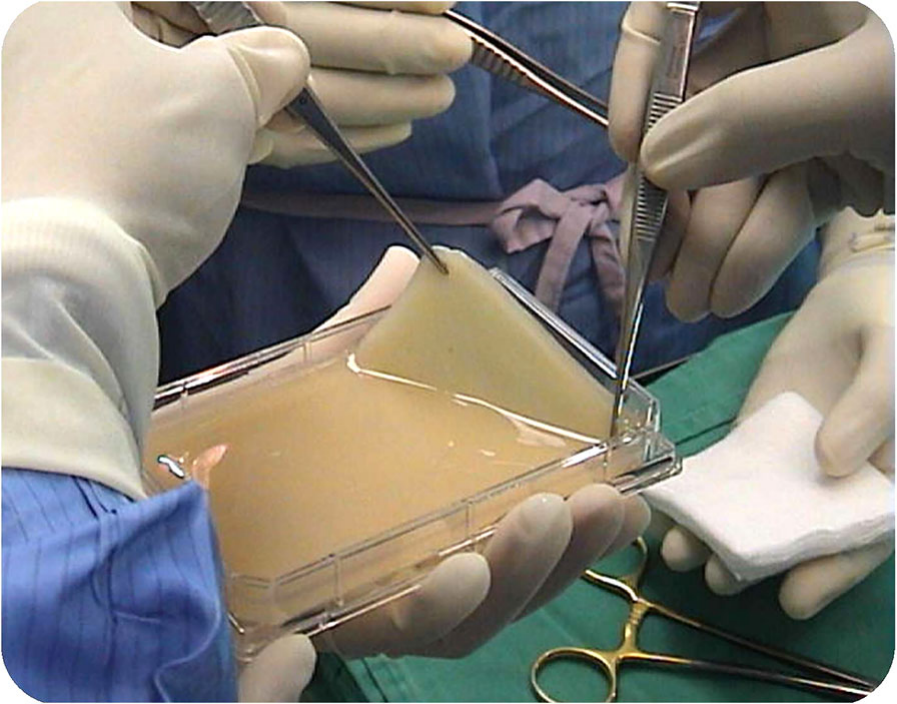
Bi-layered tissue engineered skin (MyDerm) [32–34] (produced by the Cell Tissue Technology Company from Malaysia in collaboration with the Tissue Engineering Centre, National University of Malaysia Medical Centre, Malaysian patent application No. PI20042556)

Multiple preclinical models have demonstrated the efficacy of MSC-based treatments for promoting the repair and regeneration of thermal burns and radiation exposure [18–23]. These studies have reported more expedient wound closure, decreased incidence of infection, increased vasculogenesis, increased elasticity and reduced scar formation [24–26] (Fig. 5). Lacerative injury occurring with irradiation presents a difficult treatment challenge, as has been previously reported [27]. In one study, a combination of radiation-wound injuries was generated in rats by producing an excisional wound equal to 2% of the body surface in subjects that had received 6 Gy total body irradiation. MSCs were injected directly into the wound bed and margins, and at 14 days post-injury, wound areas were approximately half the size in MSC-treated animals compared to control subjects [28, 29]. There were similarly positive outcomes found by our team [30, 31]. Clinical evidence that MSCs play a natural role in human skin regeneration has been documented. In one study, the number of MSCs circulating in the peripheral blood of thermal-burn patients was quantified and compared to the number of circulating MSCs in the blood of healthy volunteers. It was found that the percentage of circulating MSCs in burn patients was greater than 20% compared to healthy individuals, and the degree of increase correlated with the size and severity of the burn [10]. Other tissue-engineered procedures with single- or double-layered skin substitutes have been reported. For instance, “MyDerm”, a bilayered skin substitute, is currently undergoing phase II clinical trials [32–34] (Fig. 6).

**Fig. 7:**
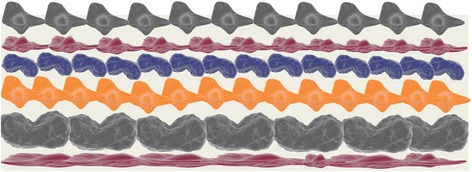
The act of tissue formation by layering of cells in a 3-dimensional scaffold to mimic the natural arrangement [36]

In 2008, the US Armed Forces Institute of Regenerative Medicine (AFIRM) began to harness stem cell technology to reconstruct new body parts, including skin. Over 2250 million dollars in public and private funds were allotted for the project’s first 5 years, with the National Institutes of Health (NIH) teaming up with three other public universities for a progressive developmental project. The results were encouraging [35]. The US Army Institute of Surgical Research (USAISR) has also developed a bio-engineered skin substitute for the treatment of burn patients with severe, life-threatening wounds. This treatment, named Engineered Skin Substitute (ESS), uses tissues made from autologous collagen-producing cells to replace the two top layers of skin. ESS was developed in conjunction with the California-based biotechnology firm Amarantus BioScience and Rutgers University [36]. The use of the patient’s own cells avoids the need for foreign substitutes, which lowers the chances of infection, avoids the use of immunosuppressants and reduces the number of surgeries required. USAISR has also investigated other treatments for severe burns, including the ReCell Spray device for skin, developed by British Avita Medicals. This technology distributes autologous healthy cells, proliferating and suspended in a physiological solution, onto wounds after the removal of dead cells. Surgeons at USAISR have also explored the science of stratigraphy, involving the layering of skin cells developed from a patient’s stem cells to grow new tissues (Fig. 7) [36].

### Sensory organ injuries

**Fig. 8:**
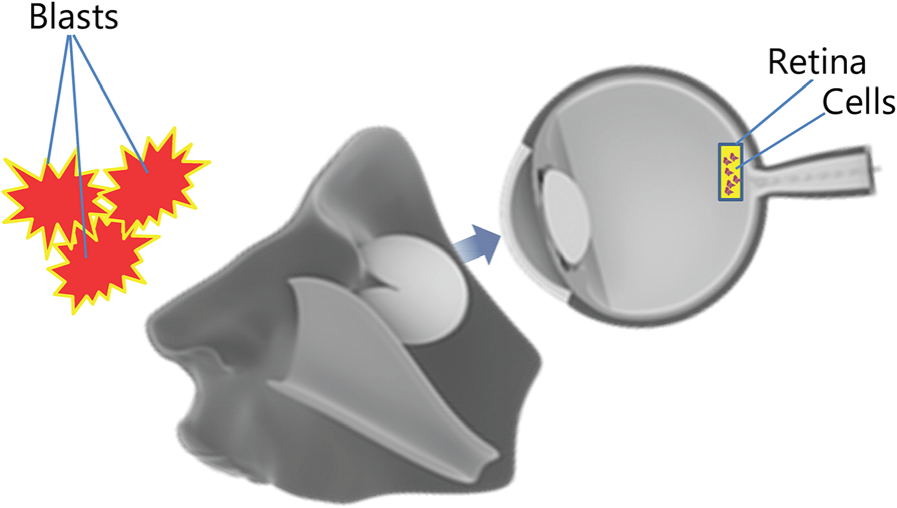
The eye and the retina showing the danger that destructive explosive devices pose to the eyes of combat personnel [27]**.** Several tissue engineering techniques have attempted to regenerate damaged retina cells

The invention of polycarbonate eye armor has ushered in an era of reduced injuries to the eye; however, recent increases in advanced explosive models have led to a reciprocal increase in devastating injuries to the eye (Fig. 8) [27]. Neurons in the photoreceptors and retinas lack spontaneous regenerative abilities, which predisposes victims to permanent loss of vision. In several instances, depending on the availability of a donor, eye transplantation can be feasible, but additional reports have suggested that native and precursor cells stand a better integrative chance with the host. Recently, it has been shown that the rod and cone cells of the retina can be produced from stem cells. Embryonic and adult stem cells have shown advances in this regard, with substantial improvement in vision restoration [37].

Auditory impairments are the most typical sensory loss experienced in a blast trauma. High-speed explosives and artillery result in mechanical and biochemical damage to the auditory system, especially to the sensory hairs. Severe injuries to hair cells in the cochlea, which lack regenerative capacity, can lead to permanent hearing loss and vertigo [38]. To date, results from experiments in animal models have demonstrated partial successful regeneration following stem cell transplantation. Moreover, comparable results have been shown with neural stem cells for hair cells in the inner ear, with the capacity to re-establish hearing, thereby providing potential tools for suitable replacements. These could be accomplished by either the differentiation or isolation of native resident cochlear cells. Nevertheless, enhancement of their functionality remains an uphill task because, apart from generating hair cells, reintegration into the network of innervation must be achieved for viable restoration of auditory function [39].

### Nervous system tissue injuries

**Fig. 9:**
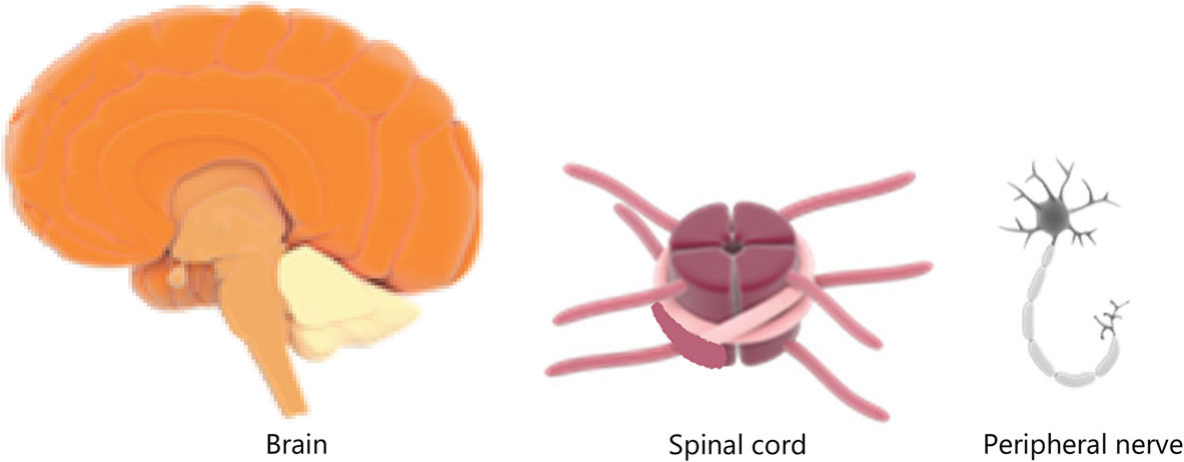
The three main tissues (brain, spinal cord and peripheral nerves) that are targets of nervous system injuries. The complexity of these structures and the integration of the specified cells poses challenges for tissue engineering

There are 3 major kinds of injuries typically sustained in the nervous system. These include trauma/concussions to brain tissues, severed spinal cords and torn peripheral nerves (Fig. 9). Projectiles, explosives, and radioactive and chemical agents pose great risks to the nervous systems of deployed troops. Most brain injuries/traumas are not reversible, owing to the delicate nature and the currently limited knowledge of brain complexity and treatment/regeneration. Injuries to the spinal cord can cause total immobility, depriving affected individuals of sensory innervation and motor responses below the affected regions [27].

According to ongoing reports, blast-induced neurotrauma has been the signature wound of present wars around the globe. This is partly due to improved protective equipment against fragmentation and penetrative weapons [40–42]. For example, up to approximately 97% of the casualties in one Marine unit in Iraq were caused by explosions. Further breakdown revealed that 65% were due to improvised explosive devices (IEDs), and 53% of injuries were to the head and neck [43].

Overall, close to 2,000 (23%) medically evacuated US troops sustained traumatic brain injuries (TBIs) during the previous war in Iraq. Closed traumatic brain injury (TBI) accounted for approximately 90% of 433 service men studied at Walter Reed Army Medical Center, highlighting that blasts are the signature injuries of contemporary conflicts [44].

Hypotheses regarding how the brain becomes pressurized as a result of blast exposures have generated much debate. Researchers have concentrated on this phenomenon, although the developmental sequence and procedures to treat the resultant pathophysiology remain unresolved [45, 46].

TBIs can be psychologically destructive, predisposing affected individuals to depression, substance abuse and personality disorders. In effect, they share many clinical features with post-traumatic stress disorder [47]. The role stem cells from the nervous system play in establishing cognitive behavior remains unclear; however, they remain a key part of neurogenesis, which is associated with the development of post-traumatic stress disorder and similar mental health problems [48]. Reports have revealed that blast 8exposure readily induces total brain microglial activation in rats between days 1 and 14 post-assault. Affected rats displayed significant activation of microglial and cell surface antigens typical of degenerative neurologic disorders, such as Alzheimer’s disease [49].

Peripheral and central nervous system trauma can pose problems on two fronts, causing both physical and psychological malfunction. Data exist that reveal peripheral nervous system trauma in approximately 20% of patients with limb injuries sustained in the Vietnam War [39]. This figure will be higher in future combat situations with increased central nervous concussions due to high energy blasts, making this topic a significant military concern [50–52]. Importantly, it is paramount to the defense forces that a returning soldier can integrate back into society with good physical and psychological states of health. Even a partial restoration of nervous system injuries is counted as a significant achievement due to the agonizing effects of neuropathic pain [27]. Patients with spinal cord and peripheral nerve system damage can benefit from varied strategies applied toward neurogenesis, remyelination and oligoprogenitors; tissue engineered cells and nerve substitutes are predicted to greatly improve recovery [27, 50].

More studies have increasingly explored the treatment of disorders associated with TBI, such as Parkinson’s disease, stroke and spinal cord injuries. A study of 7 candidates with an average age of 42 years suffering from Parkinson’s for an average period of 15 years that received autologous cultured bone marrow stem cells (BMSCs) fibroblast growth factor-2 (FGF2), at a dosage of 1 × 10^6^ cells per kilogram body weight into the sub-lateral ventricular zone, reported that 3 candidates maintained a consistent recovery in their Unified Parkinson’s Disease Rating Scale (UPDRS) “on and off” score. They also demonstrated recovery in Schwab and England, Hoehn and Yahr scores and in facial expression symptoms, episodes of gait imbalance and freezing [53]. In another report, 12 adults (8 with Parkinson’s and 4 with Parkinson’s in combination with systemic atrophy and progressive supra-nuclear palsy) were administered allogeneic BMSCs at a dosage of 2 × 10^6^ cells per kilogram body weight. Those with Parkinson’s only demonstrated continuous recovery with an average of 17.2% improvement in ‘on’ score and 31.2% improvement in ‘off’ score in the UPDRS; however, patients with Parkinson’s in combination with systemic atrophy and progressive supra-nuclear palsy had no recovery [54].

In a study of ischemic stroke, 10 acute cases received approximately 10 × 10^6^ autologous BMSCs per kilogram body weight. Of the 10 patients, 7 recovered to a Barthel Index score of greater than 90. Nine patients had a downward shift of a minimum of 1 point on the modified Rankin Scale. On the Median National Institutes of Health Stroke Scales Scores, patients decreased from a score of 13 pretreatment to 8 on the 7th day and 3 within the 6th month post-treatment [55]. In spinal cord injuries, another study revealed that MSC implantation in a murine model demonstrated anti-inflammatory effects on the injury and initiated macrophage changes from type 1 (M1) to type 2 (M2), thus enhancing tissue remodeling and scar tissue reduction upon healing [56].

Several human trials have recorded various degrees of success with MSC treatments. One study reported that out of 5 spinal cord injury patients treated with autologous bone marrow mononuclear cells (BM MNCs) and granulocyte colony-stimulating factor (GCSF) (and one control patient treated with GCSF alone), significant motor progress was observed 3 and 7 months after treatment for the MSC group. Four patients (80%) that received BM MNCs and GCSF had improved recovery from first to third grade, and one subject recovered from first to second grade. The subject who received GCSF alone remained at the first grade. There was no adverse effects observed, except for mild fever and muscle pain, which stem from the administration of GCSF [57]. Similarly, there have been several other trials with culture-expanded BMSCs that have shown marked improvement in the restoration of motor functions within a few months [54, 58].

### Musculoskeletal injuries

**Fig. 10:**
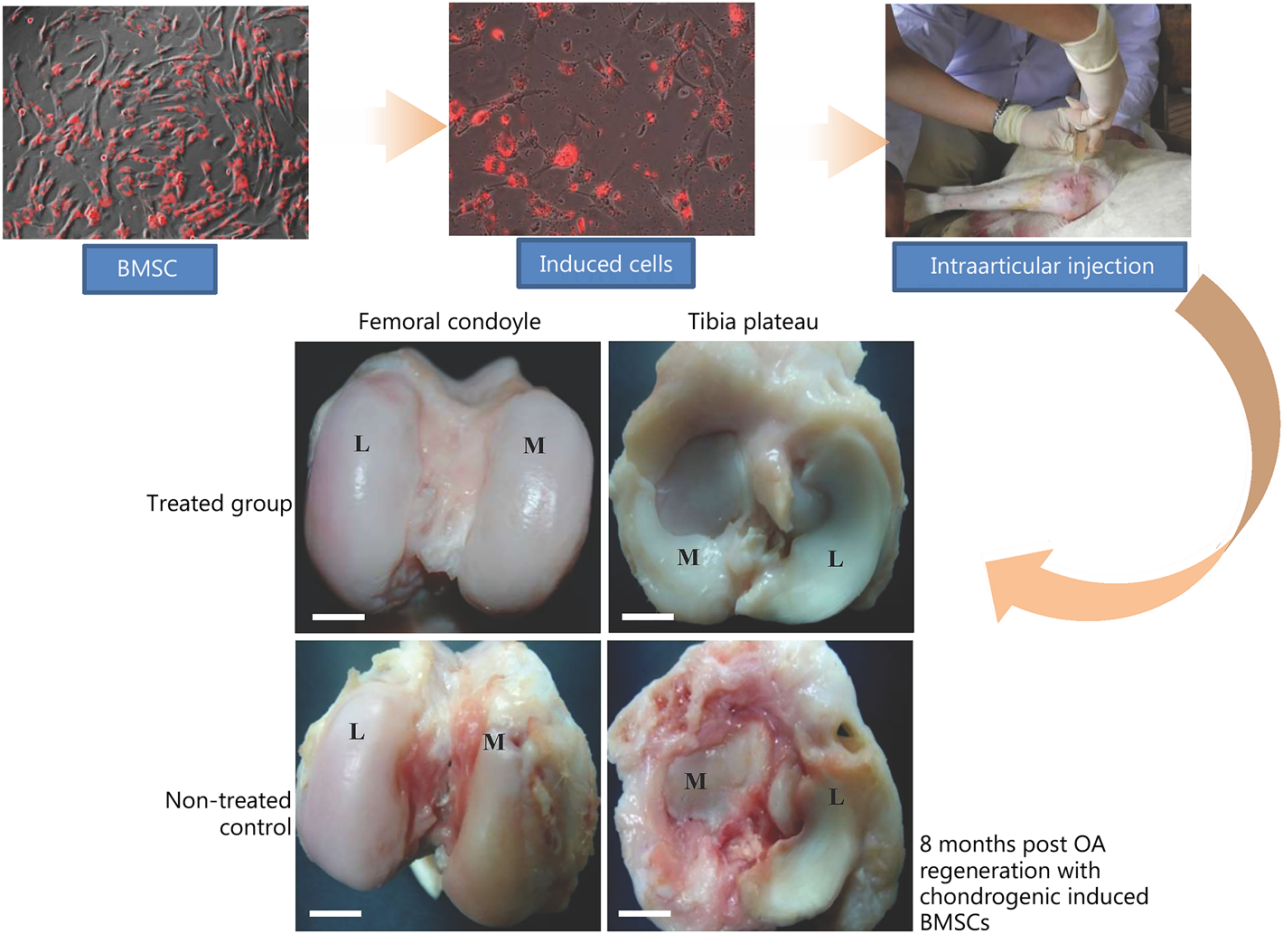
Cartilage tissue engineering showing the treatment of osteoarthritic cartilage degeneration in a sheep model with chondrogenic-induced BMSCs [62–67]. L: Lateral; M: Medial; the treated knee had remarkable improvement compared to the non-treated control

Developments in body armor, combat-ready medical personnel, enhanced wound management, improved evacuation protocols and advanced surgical technology and skills have increasingly contributed to the survival of soldiers in battle. Nevertheless, orthopedic injuries, at 65%, still remain the most common type of injury in recent conflicts [27, 59]. Almost all injuries sustained in war at least partially involve bones, cartilage, tendons, adipose tissue and muscles, making them a priority area of defense medicine. Musculoskeletal injuries limit a soldier’s ability to perform due to excruciating pain caused by fragments of shrapnel and bullets and accompanying inflammation caused by blasts [60]. A high incidence of heterotopic ossification associated with injuries from blasts is believed to be the result of local stem cell mobilization. This causes poor prosthesis fitting, limits joint mobility and often necessitates further surgery for removal. It has been reported that up to 15% of long bone injuries sustained in war become chronically infected with osteomyelitis [61]. Stem cells have been proven to be useful in augmenting responses to infections, as well as improving the ability to treat chronic ailments such as osteomyelitis and osteoarthritis. Extensive work has been done on bone and cartilage regeneration in osteoarthritic models (Fig. 10) [62–67]. Conventional management of these conditions often requires multiple surgical procedures over many years and can still result in amputation or death [27, 68].

Another major focus of musculoskeletal injuries is compartment syndrome, due to internal muscular wounds sustained from blasts and other trauma. This results in rapid inflammation in the upper or lower limbs, compressing the arteries, veins and nerves; if there is no immediate treatment, myopathy can ensue that necessitates amputation. According to earlier reports of US troops, the Iraq war resulted in more than 800 amputations through similar conditions [69]. With their proven efficacies, administration of stem cells can salvage this situation. Furthermore, segmental bone defects represent ongoing challenges in the management of extremity traumas related to high-energy blast injuries. Several techniques have been used in civilian populations to treat bone defects, with varying degrees of success. Vascularized bone grafts have been successfully employed in civilian settings for the treatment of osteonecrosis of the femoral head and in the reconstruction of segmental defects resulting from trauma, infection or tumor resection [70–73].

In a study that evaluated the osteogenic potential of composites of chitosan-β-tricalcium phosphate for regenerating long bone defects, stem cells with an injectable form of the composite were used as a treatment strategy. It was found that implantation of stem cells with a carrier composite functioned efficiently in repairing defects in long bones. Histological sections showed clear micro and macro blood vessels. Nutrient vessels extended into the endostea and periostea; in addition, Haversian canals were observed in the middle, internal and external lamellae, with blood vessels reticulating along Haversian and Volkmann’s canals [74].

In a previous annual American Academy of Orthopaedic Surgeons symposium (Extremity War Injuries: EWI), jointly organized by the US Department of Defense and the Orthopaedic Extremity Trauma Research Program (OETRP), the management of severe injuries sustained primarily as a result of blasts associated with military operations in the global war on terror, as well as challenges and opportunities in the management of combat-related musculoskeletal injuries, was discussed. It was recommended that understanding of the proper organization of soft tissues and musculoskeletal tissues was required for locomotion and normal skeletal function. It was proposed that more efforts be focused on understanding the properties of the individual components of these tissues to enhance tissue engineering approaches to maximize their functions within the limb [72, 73].

Generally, tissue engineering approaches within the musculoskeletal tissues have shown promise, and MSC isolations from every tissue have necessitated more precise naming, including bone marrow stromal cells, adipose-derived stem cells, and others [75]. These have all shown an ability to differentiate into principal cell types of mesodermal origin, as well as other lineages. Many preclinical and clinical studies have shown that a mixture of cells and scaffolds (biomaterials) likely represents the ideal technique for structural repair and regeneration of musculoskeletal tissues [76].

### Circulatory/pulmonary tissues

**Fig. 11:**
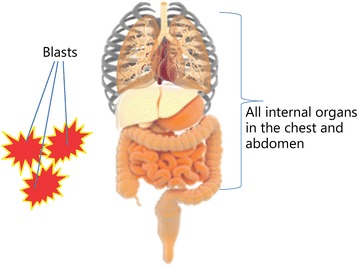
Tissues and organs exposed to blast over-pressurization [77]. These including lungs, heart, liver intestine and internal organs situated in the thoracic cavity are all in danger from this type of injury

A new type of injury, blast over-pressurization, also known as blast lung, has been identified among blast victims. These injuries are composed of arterial air emboli, pulmonary hemorrhage and contusions of the lung (Fig. 11) [77]. Approximately 70% of severely wounded soldiers have some degree of pulmonary injury, though initial diagnoses and clinical presentations can vary widely [27]. In civilian settings, stem cells from pulmonary tissues have been extensively studied with regard to their connection with lung cancer. Few studies have determined the mechanisms and the regenerative capacities of these cells for use in post-traumatic war-time trauma. Regenerative measures geared toward pulmonary fibrosis and other lung injuries using MSCs or ESCs will certainly benefit defense medical treatment of blast lung [78].

In hemorrhage and blood loss, emerging technologies have aided scientists in developing artificial blood that could treat wounded soldiers in far-flung battle fields. This procedure, named “pharming,” was launched by the US Pentagon for its Defense Advanced Research Project Agency (DARPA) in 2008 [3]. Mass quantities of genetically engineered red blood cells were created from umbilical cord stem cells with a machine that mimics the three-dimensional structure of hematopoietic activities in bone marrow. Researchers from the innovating firm “Arteriocyte”, relying on a technique developed at Johns Hopkins University, were trying to grow large batches of stem cells when they realized that the proliferating conditions had caused stem cell differentiation into pro-erythroblasts. This initially caused frustration, but the researchers subsequently realized that they had unintentionally found a smart way to produce new blood. With an initial 1.95 million dollars available for the project, they provided O-negative blood samples to the US FDA for evaluation and endorsement [3, 79–81].

### Genital/testicular injuries

The US military has funded research into lab-grown testicles for soldiers whose battlefield injuries have left them unable to conceive children. This Pentagon-backed research at the Wake Forest Institute for Regenerative Medicine in North Carolina could fundamentally alter the prognosis of men with injuries impairing their procreative capability [4, 82]. Though certain genital injuries could be prevented by protective gear used by troops, such as a Kevlar plate that covers the groin, the catastrophic effects of explosions could still be observed in hundreds of soldiers. It was estimated that approximately 500 soldiers wounded in Iraq alone sustained injuries that rendered them unable to bear children [83]. Tissue engineers have managed to reconstruct intact testicles using autologous stem cells from soldiers. These testicles, which create sperm, can be inserted into donor patients [84]. Additionally, the British Army introduced alternative steps of storing sperm from would-be deployed soldiers, in case they suffered severe genital injuries in the course of duty; hence, soldiers had the option of freezing their sperm cells prior to deployment [85].

Other works that have given hope to men and women with traumatic genito-urinary injuries from war include tissue engineering of a penis, tissue-engineered autologous urethras and tissue-engineered autologous vaginas [84, 86, 87]. A pilot cohort study was jointly carried out by Wake Forest University and Hospital Infantil de México Federico Gómez on 4 teenage girls (13-18 yrs.) who received autologous tissue-engineered vaginas with an average follow-up period up to 8 years. It was concluded that the engineered organs showed normal structural and functional characteristics [87].

## Overcoming threats posed by acute radiation syndrome (ARS)

Strategies to solve potential threats posed by the incidence of ARS include, but are not limited to, enhancement of hematopoietic recovery and facilitation of healing of the gastro-intestinal tract and the skin.

Hematopoietic complications are among the main causes of death upon exposure to radiation ranging from 1 to 10 Gy [11]. The resulting complications include severe immuno-suppression, anemia and low platelet counts from reduced production by the bone marrow [11]. It has been demonstrated that the mean lethal whole-body dose of radiation that can kill half of the victims in less than 2 months is approximately 4 Gy. In a situation where patients receive appropriate medical attention with intravenous infusions, antiemetics, analgesics, antibiotics and transfusion of fresh compatible blood, casualty rates are reduced. Current management guidelines include short-term cytokine therapy with GCSF and granulocyte-macrophage colony-stimulating factor (GM-CSF) to facilitate hematopoietic recovery [88]. Mesenchymal stem cells are known to secrete hematopoietic cytokines, including leukemia-inhibiting factor, G-CSF, and GM-CSF [12]. Several reports have indicated that the addition of stem cell therapy to standard treatment can promote more rapid recovery of the hematopoietic system due to the inherent capabilities of the stem cells. Preclinical studies have evaluated the combination of hematopoietic stem cell (HSC)-MSC co-transplantation in hematopoietic recovery, with promising results. This has been translated into the clinics as well. One proven successful clinical technique is the co-infusion of HSCs-MSCs following ablative radiation or chemotherapy for the treatment of both malignant and non-malignant disorders [12].

In another study, patients with serious burns from radiation, who were not benefitting from a conventional treatment course, were transplanted with 5 grafts of autologous cultured BMSCs. It was observed that these patients experienced significant decreases in their blood levels of C-reactive proteins for over 100 days post-stem cell treatment. They further observed overall reductions in pain, a disappearance of tissue necrosis and soft tissue regeneration, especially on the arm [89].

For the advancement of civilian and defense responses to possible nuclear events, the US Department of Defense partnered with Osiris Therapeutics in the development of “Prochymal” for possible mass production. Prochymal, an allogenic stem cell therapy, was first approved by Canada for acute graft-vs-host disease (GvHD). MSCs derived from pooled bone marrow from healthy adults between the ages of 18 and 30 years were isolated, cultured, packaged and stored frozen until needed. These could be administered post-exposure or at the onset of symptoms, eliminating the need for a predefined treatment. The long-term storage capability of Prochymal makes stockpiling it for a mass-health event feasible [90, 91].

Patients who are exposed to a level of radiation greater than 7 Gy will experience complications characterized by electrolyte imbalances, anorexia, malaise, dehydration, fever and severe diarrhea. The main root cause of the injuries to the gastro-intestinal tract is linked to sudden depletion and death of intestinal crypt cells, which are precursors to the epithelial layer cells that normally replenish them on a regular basis. This depletion causes a breach in the normal gastro-intestinal barrier and predisposes the subject to life-threatening infections. People diagnosed with this condition have low probabilities of survival; to date, there has been no specific medically approved response to this condition [10].

Several studies have reported that MSC transplantation can aid regeneration and restoration of the intestinal epithelium by nurturing continuous replication of the remnant stem cells within the crypt. It is presumed that the mechanism by which MSCs elicit this support is by local secretion of cytokines and chemotactic signals that induce the migration of progenitor cells to injury sites. Animal studies of abdominal complications of ARS have shown increased survival with MSC transplantation, together with the regeneration of cells from the internal crypt, restitution of the stem cell bases, and increases in xylose absorption [9]. The serum levels of intestinal radio-protective agents (recombinant human R-Spondin 1(R-Spondin-l), FGF2, platelet derived growth factor (PDGF) and KFG) increased in concert with anti-inflammatory cell signals, whereas inflammatory cell signals were decreased [28]. Furthermore, reports of allogeneic MSC infusion systematically administered to an accidentally over-irradiated patient who had prostate cancer and developed induced colitis showed reduced pain, diarrhea, hemorrhage, inflammation, and fistula formation [92].

## Utilization of stem cells in biosensors increases safety

In modern warfare, the increased threats posed by chemical and biological weapons underscore the importance of the early detection of the presence of these agents [93].

Due to their plasticity, stem cells have helped to diminish the concerns posed by various non-physical weapons, as well as to better appreciate their acute or extended effects via screening. These results have helped to develop biosensors with greater precision for detection [94].

In preparation for risks to deployed forces, the US DARPA and several other military, academic and private institutions have developed classes of engineered tissue-based biosensors (TBBs) and biologically based biosensors with the capacity to detect almost any environmental chemical or biological hazard that can inflict acute (irritant, performance incapacitation or death) or chronic (organic illness, cancer or genetic) health consequences on military or civilian populations. One example of this is the US Army Center for Environmental Health Research (USACEHR) ventilator system [95].

The major agents of concern for deployed troops primarily include those with the capacity to be weaponized, including (a) neurotoxic agents (mustard, sarin, soman and VX agent (O-ethyl S-2-di-isopropylamino ethyl methyl phosphonothioate)), (b) occupational chemicals (isocyanates, toluene and benzene), (c) environmental toxins (botulinum toxin), (d) industrial wastes, (e) military reagents and materials (propellants and refrigerants), and (f) combustion byproducts (carbon monoxide, cyanide and polycyclic aromatic hydrocarbon (PAHs)). These agents can pose threats individually, in simple mixtures, or as complex mixtures with the potential for addition, synergistic or antagonistic reactions [95, 96].

TBBs can be categorized into two groups: a) those with detector elements derived from higher animal cells and b) those with detector elements derived from simple organisms, which are occasionally genetically engineered for specificity (e.g., bacteria and yeast) [96].

They can detect significant concentrations of well-known biologically active agents or new potential lethal agents and send early warning signals to deployed troops. Biologically based sensors utilize biological components as sensing elements, which are compatible with chemical, electrical or optical transduction [97].

Their detector elements include mammalian brain tissues, neuronal and glial cell cultures, spinal cord cultures, cardiomyocyte cultures, hepatocyte cultures, fish scale chromatophores and genetically engineered bacteria or biologically active cell processes [27, 95, 98–101].

Furthermore, military and civilian jet crews are continually in contact with higher doses of natural radiation from galactic cosmic rays at typical jet altitudes of 6.1 to 18.0 km. Exposure often occurs from sporadic solar flare activities, especially at higher supersonic jet altitudes. Isotropic particle fluence rates of primary cosmic rays, comprising 90% protons, 9% alpha particles and 1% heavy nuclei from carbon to iron, are derived from stellar flares and coronal mass ejections, pulsar acceleration, supernovas and explosions of galactic nuclei [101, 102]. In the 1990s, the International Commission on Radiological Protection (ICRP) recommended that jet crews be classified as occupationally vulnerable to radiation. Further reports from prominent Canadian airlines have determined that galactic exposure to aircrews is comparable to that listed in the National Dose Registry [103]. Other bodies, such as the European Union Basic Safety Standard Directive, the United States Federal Aviation Administration (FAA), and Transport Canada, among others, have developed regulatory policies in line with the ICRP recommendations for aircrew exposure monitoring [104].

Over the years, tissue-equivalent proportional counters (TEPCs) have been used to measure the ambient dose equivalents of the cosmic radiation exposure of aircrews during a solar cycle. Measurements performed at the International Space Station (ISS), ground-based neutron monitoring, Geostationary Operation Environmental Satellite (GOES) data and data from transport code analysis have been used to propose an empirical model for estimation of aircrew exposure during solar particle events. These data have been compared to results obtained during recent solar events [101]. With recent developments in tissue engineering, it is believed that engineered tissues are better suited for re-evaluating these policy-making measurements for accurate data concerning the effects of galactic cosmic rays and solar flares on native tissues.

## Discussion

Organ reconstitution and tissue regeneration have been the primary goals of tissue engineering in defense medicine. Virtually every tissue in the body is of military importance due to the diverse nature of injuries associated with war [10]. More than 50,000 returning US troops were wounded in Afghanistan and Iraq alone; the majority of these injuries were inflicted by improvised explosive devices. Hundreds of soldiers lost their hands and legs [4, 72, 73, 82].

In war, it is difficult to have localized damage to a single tissue type from combat; injuries always reflect a mix of integrated wounded tissues. In the past, several techniques have been used to treat these defects, with varying degrees of success [27, 70, 71].

Physicians at the United States Walter Reed Army Medical Center have reported that each war has its own characteristic wounds. World War I produced damaged lungs from poisonous gases, World War II inflicted cancers as a result of radiation from atomic bombs, and the Vietnam War produced skin disorders from Agent Orange exposure. In recent conflicts, blast-induced TBI has emerged as a signature wound [105].

Tissue engineering has propelled the concept of regenerative medicine into promising realities. To properly engineer complex tissues, stem cells will need to be incorporated into biomaterials. This was not discussed in detail here, but current developments in scaffolds have been captivating and impressive [5, 6, 27].

The human body routinely regenerates liver and bone marrow cells. Fundamentally, all body parts, tissues and organs have a natural repository of cells that are available for replication when an injury occurs. Initially, efforts were focused on skin substitutes for treating burns, but an increasing number of tissue types, biomaterials and scaffolds used as delivery systems are now being engineered. Notable results have included tissue-engineered bone, cartilage, blood vessels, liver, muscle, and even nerve conduits [5, 7, 12, 23, 28, 34, 63, 64, 66, 67, 106–108].

There are approximately 300 institutes in the USA and more than 80 in Europe that have embarked on stem cell, tissue engineering and regenerative medicine projects, both for military and civilian purposes [109]. Several of the notable stem cell companies around the world, mainly privately owned, that are at the forefront of these technologies are listed in Additional file 1: Appendix. Based on DARPA’s initiative into blood pharming, it is believed that fresher blood is superior to older blood because it carries more oxygen and speeds recovery. Normally, it may take up to 2 weeks for donated blood to reach soldiers who need immediate transfusions, but with a synthetic machine, DARPA could churn out liters of blood on-demand for injured soldiers. Furthermore, if this works adequately for the military, it is expected that it should also work for domestic hospitals, which pay increasingly more expensive bills for blood that is in short supply [3].

The United States Army Medical Research Command (USAMRC) has focused on musculoskeletal tissue regeneration over the last several years. At the project initiation, it was acknowledged that recent wounds are more horrific; although death rates from injuries have dropped, more veterans face lives with broken limbs, which have increasingly called for efforts focused on regenerating them. This will help keep veterans on active duty and enable them to have normal lives [110]. The United States AFIRM has proposed the restoration of function to limbs that would otherwise be amputated, as well as pursuing hand and limb transplantation in worst-case scenarios. Furthermore, they initiated a long-term project to regenerate and re-grow wounded soldiers’ limbs, arms and other extremities. Another project pioneered included MSCs for bone regeneration via the introduction of cells into poly-carbonate scaffolds that were free of bisphenol-A, a potential endocrine disrupter [111].

Since 2012, apart from collaborations with notable academics, AFIRM collaborated with other tissue regeneration companies, such as Organogenesis, in Canton, and Intercytex, in Woburn Massachusetts, to create an “off-the-shelf” composite skin substitute. One of their most conventional projects was to optimize a skin-grafting technique with more modern skin spraying and printing technologies [112]. Many returning wounded soldiers are burnt so extensively that their own skin is insufficient for grafts or harvests for culture. Allogeneic skin and skins from cadavers are prone to rejection; hence, similar to the British Army’s development of storage alternatives for sperm of would-be deployed soldiers for the generation of new testicles, AFIRM had envisioned a project of storing skin samples from every soldier in dangerous areas so that the moment a soldier is injured, people back at the main Army medical center could start growing a required graft [83, 112].

In Russia, where tissue engineering was developed for the Defense Ministry in the era of the Soviet War in Afghanistan, scientists have attempted to use stem cells to cure psychological war traumas (PWT). The use of stem cells for PWT has progressively developed in Russia. In June 2014, the Russian Ministry of Defense announced its decision to continue research into PWT. A new scientific division within the Military Medical Research was formed and was subsequently divided into three units. These included the Biological-pharmaceutical, Medical-prophylactic, and Engineering-technical units. At first, they worked on the creation of a stem cell bank for soldiers who operated in risky assignments and in dangerous areas [109]. In 2013, the Human Stem Cell Institute (HSCI) opened the Genetic Center in Moscow; among the diseases it targeted were congenital immunodeficiency, Krabbe disease, Omenn syndrome, Diamond-Blackfan syndrome, and Fanconi anemia. They also developed Neovasculgen, designed to treat patients suffering from critical limb ischemia. With a global estimate of more than 200 million people suffering from this ailment, the discovery of Neovasculgen was considered a great milestone, particularly in countries such as Russia, where it has been the single largest cause of amputation, with over 500 cases per 1 million people per year [113].

As part of Britain’s preparations for a possible nuclear attack, their military partnered with the Pentagon on the production of Prochymal, which is believed to rejuvenate blood and repair damaged skin. Both the United States and the British government proposed investing millions of dollars in its purchase after approval from the FDA [114]. The British Military Chemists of the University of Sheffield have also developed a tissue-engineered technique that detects and contains chemical weapons. Their research was focused on organophosphorus chemical weapons that attack nerve cells, such as sarin and soman. With incidents of chemical agents deployed by terrorists, such as the doomsday cult in the Tokyo subway sarin attack, and the deployment of chemical arsenals by both the Syrian government and the rebels in the on-going conflicts in Syria and Iraq, there is a greater need for early detection and dispersal [115, 116]. Recently, scientists from Newcastle University have developed, for the first time, technology to put stem cells into plaster bandages, which helps heal wounds. The stem cells are encapsulated in an alginate gel made from seaweed that offers them protection from the environment, similar to frogspawn. It was found that the cells could be viable for up to 3 days. This new invention could be vital to the military for the rapid treatment and healing of injuries, burns and lacerations sustained in the line of duty [117].

In Malaysia, there is a pioneering center at the National University of Malaysia Medical Centre and other offshoots, among which is the Bio-artificial Organ Unit at the National Defence University, whose aim is to engineer bio-artificial organs for military medicine [118]. With the recent chemical VX agent attack at the Kuala Lumpur International Airport-2, [119, 120] there is a critical need for more research and development of TBB biosensors and detectors for such lethal agents.

In China, there has been immense focus in both civilian and military research on stem cells and tissue engineering programs since the early 2000s. The application of stem cells in this area has mainly focused on hematopoietic stem cells and the use of MSCs in hematological diseases, vascular diseases, and diabetes mellitus. Among the major laboratories and centers for tissue engineering and stem cell biology are the Fourth Military Medical College Center for Tissue Engineering and the Institute of Basic Medical Science Academy of Military Medical Sciences. Several of the approved clinical trials that are monitored by the State Food and Drug Administration (SFDA) include: 1) autologous bone marrow mesenchymal stem cells for injection (X0407487); 2) recombinant stem cell factor for injection (CSL01037); 3) umbilical cord blood cell progenitor cells for injection (X0404120); 4) umbilical cord blood red blood cell precursors for injection (X0404119). All these were performed by the Institute of Transfusion Medicine, Academy of Military Medical Sciences of People’s Liberation Army [121]. In 2007, the SFDA approved ActivSkin, the first tissue-engineered skin substitute developed by the Fourth Military Medical University, Xi’an. In 2009, a sweat gland was regenerated using BMSCs, which has been applied to more than 30 cases with a follow-up period of approximately 4 years [122]. In 2010, a bone repair scaffold developed by Fuzhai Cui from Tsinghua University was approved for clinical use, and estimates show that it has been used in more than 30,000 patients [121]. In 2013, in a trial that was of military importance, transplantation of umbilical cord stem cells was performed in patients with post-traumatic brain syndrome. In approximately 40 patients who received treatment at random, the results revealed improved neurological function [123]. Apart from these products, already tested in human, Chinese scientists continue to make advances, including the development of tissue-engineered decellular dermal matrices, skin-containing adipose layers, pigmentation, capillary-like networks, hair follicles, dermal equivalents, engineered tendons, cartilage, bone, neural tubes, and more [121].

India is among the ten top countries deeply involved in tissue engineering and regenerative medicine research. The Indian stem cell industry, including both military and civilian research, was estimated to be worth approximately $500 million in 2010, with a projected annual growth rate of 15%. These research areas were originally a sub-segment of the biotechnology industry. The leading research institutes in India include the National Centre for Biological Sciences (NCBS), Bangalore; the Centre for Cellular and Molecular Biology in Hyderabad; the L.V. Prasad Eye Institute, Hyderabad; the Centre for Stem Cell Research, Vellore; the National Brain Research Centre, New Delhi; and the National Centre for Cell Science, Pune [124]. Several key private stem cell companies are listed in the Additional file 1: Appendix.

These stem cell companies and institutes have recently pursued trials on such diverse ailments as strokes, spinal injuries causing partial paralysis and cerebral artery infarction, among others [125]. In 2009, a clinical trial to determine whether patients with subacute ischemic stroke would benefit from an infusion of the patient’s own bone marrow-derived stem cells was initiated in collaboration with two military institutions; the Army Hospital for Research and Referral, New Delhi and the Armed Forces Medical College, Pune. In this study, 30-500 million autologous BMSCs were given intravenously to patients with acute ischemic stroke of less than 30 days’ onset, following the hypothesis that there would be a reduction of infarct volume and improvement of neurological function of patients with subacute ischemic strokes. The results, published in Stroke 2014, were impressive [124]. In 2015, one of the private companies, Nutech Mediworld, based in Green Park in south Delhi, announced the complete treatment of a senior army officer who suffered a severe spinal injury causing partial paralysis after successful injection of isolated human embryonic stem cells. They reported that the therapy had no known side effects and, unlike other organ transplants, did not require any immuno-suppressants [126].

**Fig. 12:**
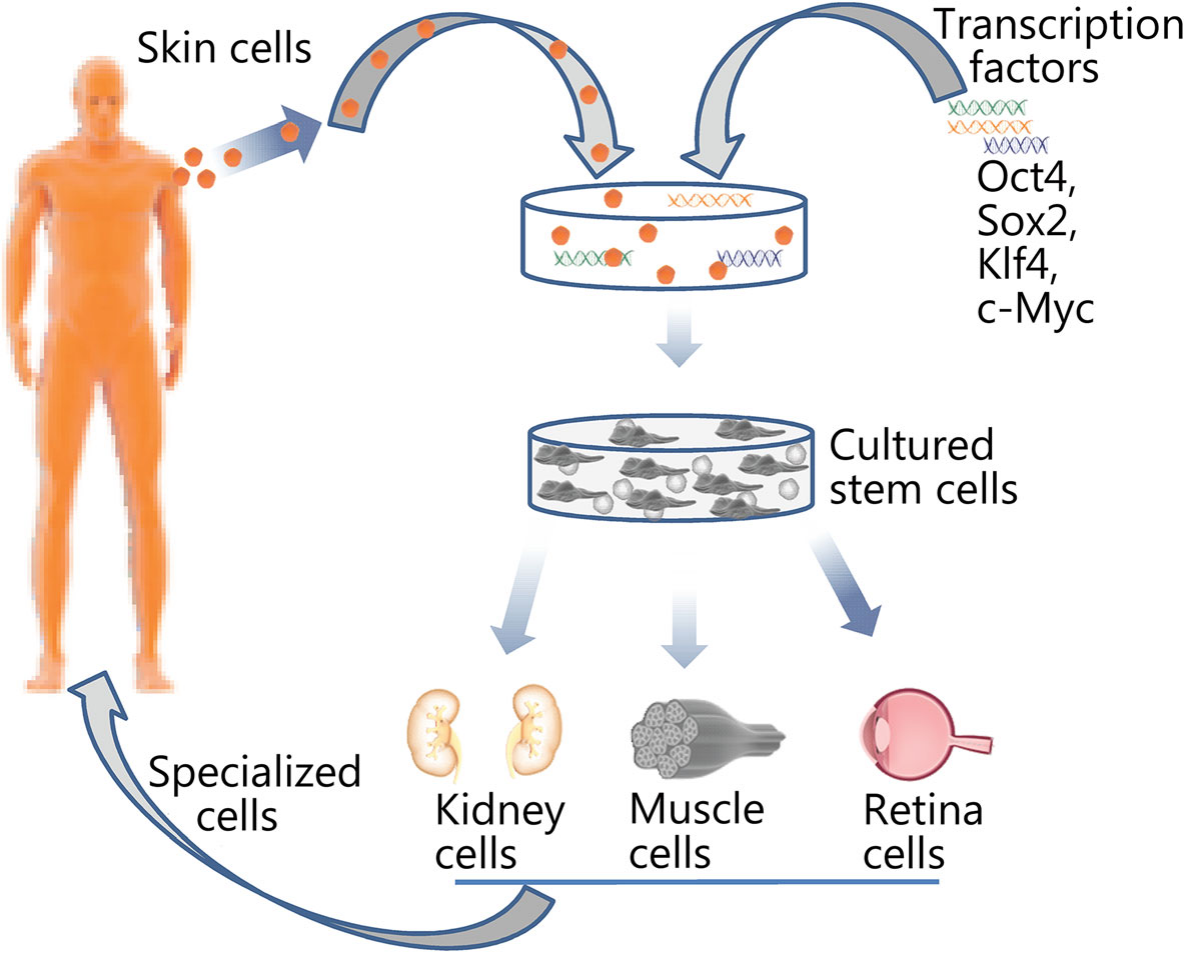
Schematic diagram of the creation of iPSCs from skin cells

In Japan, there has been enormous progress with the use of stem cells and tissue engineering technology. The invention of induced pluripotent stem cells (iPSCs) in Japan 10 years ago served as a game changer for the study and potential therapeutic use of embryonic stem cells, which have attracted the wrath of moralists as well as religious evangelicals [127]. Unlike embryonic stem cells from the blastocyst, the iPSCs technique reprograms skin or other adult cells with genes or proteins to make a similar culture of pluripotent stem cells. In recent years, the Japanese government has expanded its commitment to military and civilian research using stem cells, especially iPSCs, in order to maintain their global lead in the field co-pioneered by their scientist Shinya Yamanaka. Early clinical applications from 2013 ranged from generating neurons to replace failing brain cells in Parkinson’s disease patients and rescuing retinal cells of aged people developing progressive blindness. Recently, a private company from Japan, “Megakaryon”, reported the mass production of platelets from iPSCs. This is a critically important discovery for the military. Platelets are critical for accident or trauma victims, as well as during cancer treatments and organ transplantation. A clinical trial is slated to start in 2018. It is proposed to begin in Japan, followed by the US and later in Europe. If all goes well, the products may be in full medical use by 2020 [127]. Other notable Japanese accomplishments with iPSCs include: 1) the first successful skin-to-eye stem cell transplant in humans, where stem cells derived from a patient’s skin were transplanted into her eye to partially restore lost vision; 2) allogeneic transplantation of iPSCs, where a Japanese man in his 60s became the first person to receive cells derived from induced pluripotent cells donated by another person and the surgery is expected to pave the way for more applications of iPSCs technology; 3) the production of a complete, fully functional tooth by scientists from Queensland University of Technology from iPSCs implanted into the kidney of a mouse. This fully functional, complex cell tissue represents the key vital step scientists have been waiting for; it supports the hope of growing a fully functional limb or other vital organ in the future [128–132] (Fig. 12).

In Australia, which has been among the world’s leading countries in stem cell research and tissue engineering, significant legislation has been put in place to keep hES cells, somatic cell nuclear transfer and therapeutic cloning out of active research areas. In 2010, the Australia spinal cord injury network (Spinal Cure Australia), in collaboration with the US company Asterias Biotherapeutics, tried to develop stem cell technology that had successfully restored some movement in quadriplegics. This technology induced some recovery in arm and hand function in a patient paralyzed from the neck down after a low-dose injection of stem cell into the patient’s spinal cord. With a new protocol, when a higher injection dose was administered, there was a more robust return of arm and hand function, together with motor function, within 3 months following cell injections [133]. Recently, the Aikenhead Centre for Medical Discovery, based at St Vincent’s Hospital in Melbourne, reported the use of a 3D printer as part of a potential solution for the prevention of osteoarthritis. The study group hopes to prevent the onset of osteoarthritis by printing live cells onto damaged cartilage, bones and tendons. This may also be applicable in other organs. The biopen, a 3D-printing pen filled with stem cell “ink”, has successfully been tested to repair sheep models of knee injuries. This could benefit not only osteoarthritis patients or athletes but also a myriad of pathologies of cartilage degenerations. Its success would certainly decrease the number of patients with OA in the future [134].

Despite the advances described above, there are certain limitations with tissue engineering technology in defense medicine. One major concern is the cost of production, which could be discouraging. Over the years, the US Defense Ministry has invested millions of dollars in various projects that the Pentagon believed could 1 day help wounded veterans regain normal lives, but many expected products are far from deployment [3]. Another challenge is the complex nature of every individual tissue/organ. For instance, extensive work has been done in the central and peripheral nervous system, but not many clinical applications are yet available, particularly for cases of trauma [135]. In genital/testicular regeneration, the most significant obstacle is size because the lab-grown testicles to date are nearly microscopic. The ultimate goal is to grow full-sized testicular tissue, expand the cells and put them back into the patient. However, for a whole testicle, there is a very rich blood-vessel supply, as seen in many other intricate tissues in the body [4, 82]. Additionally, the secrecy policy of most countries’ military research is limiting. AFIRM performs extensive research, but its findings may never be released to the general public. They consider their research to not be primarily for the sake of publication; they instead intend to benefit their own soldiers. Hence, no matter the extent of the contributions from civilian tax payers, the review and dissemination of the research findings will remain opaque [110].

Despite these challenges, there is the belief that scientists will 1 day select cells from human donors and process them to re-grow new tissues and organs within the same patient, thus avoiding rejection. Techniques are being developed by various defense ministries that can make new muscle and tendon and repair extremities, such as fingers, noses and ears [3, 4, 60].

## Conclusion

With the present progress of the use of stem cells in tissue engineering applications for defense medicine, future injuries in warfare may never be the same. Current developments in military medicine are already saving the lives of soldiers who would have died from their injuries in previous conflicts. This demonstrates that yesterday’s sure deaths have become today’s wounds; if extrapolated based on current trends, today’s permanent wounds may very well become tomorrow’s bad memories, when lost hands and feet can be successfully regenerated.

## Additional file

Additional file 1: Notable stem cells and tissue engineering companies around the globe. (DOCX 30 kb)

## Abbreviation

AFIRM: Armed Forces Institute of Regenerative Medicine
ARS: Acute radiation syndrome
BMMNCs: Bone marrow mononuclear cells
BMSCs: Bone marrow stem cells
DARPA: Defence Advanced Research Project Agency
ECM: Extracellular matrix
ESCs: Embryonic stem cells
ESS: Engineered skin substitute
EWI: Extremity war injuries
FAA: Federal aviation administration
FGF2: Fibroblast growth factor 2
GCSF: Granulocyte colony-stimulating factor
GM-CSF: Granulocyte-macrophage colony-stimulating factor
GOES: Geostationary operation environmental satellite
GvHD: Graft -vs- host disease
HSC: Haemapoetic stem cell
HSCI: Human stem cells institute
ICRP: International commission on radiological protection
IEDs: Improvised explosive devices
iPSCs: Induced pluripotent stem cells
ISS: International space station
KFG: Chromosome 15q21.2 that encodes Fibroblast Growth Factor-7
MSCs: Mesenchymal stem cells
NCBS: National Centre for Biological Sciences
NIH: National Institute of Health
OETRP: Orthopaedic Extremity Trauma Research Program
PAHs: Polycyclic aromatic hydrocarbon
PDGF: Platelet derived growth factor
PWT: Psychological war traumas
R-Spondin1: Recombinant human R-Spondin 1
SFDA: State Food and Drug Administration
SSG: Split skin graft
TBBs: Tissue-based biosensors
TBI: Traumatic brain injury
TEPC: Tissue equivalent proportional counter
UPDRS: Unified Parkinson’s disease rating scale
USACEHR: US Army Center for Environmental Health Research
USAISR: US Army Institute of Surgical Research
USAMRC: United States Army Medical Research Command
