# Supplementary material for: Application of stem cells in tissue engineering for defense medicine
Source: Mil Med Res. 2018 Feb 26;5:7. doi: 10.1186/s40779-018-0154-9 (PMC6389246; doi:10.1186/s40779-018-0154-9)
Supplement: Supplementary file 1 — Notable stem cells and tissue engineering companies around the globe. (DOCX 30 kb) [file 40779_2018_154_MOESM1_ESM.docx]

| **SN** | **Location** | **Company** | **Special field** |
| --- | --- | --- | --- |
|  | **Australia** |  |  |
| 1 | Armadale, Vic | Cynata Therapeutics | Stem Cell Manufacturing Technology |
| 2 | East Melbourne | Cell Therapies | Cellular Therapies |
| 3 | Melbourne Vic | Living Cell Technologies | Regenerative Medicine |
| 4 | Melbourne VIC | Mesoblast | Regenerative Medicine |
| 6 | Pymble NSW | Regeneus | Stem Cell Therapy |
| 7 | South Melbourne Vic | Ascend Biopharceuticals | Immunotherapy |
|  | **USA** |  |  |
| 8 | Alameda, CA | [Orthocyte (BioTime)](http://orthocyte.com/) | Cellular Therapies |
| 9 | Beverly Hills, CA | [Capricor Therapeutics](http://capricor.com/#/home) | Stem Cell Heart Treatments |
| 10 | Beverly Hills, CA | [Neurogeneration](https://neurogeneration.com/) | Autologous therapy |
| 11 | Carlsbad, CA | [International Stem Cell](http://www.internationalstemcell.com/) | Proprietary Stem Cell Induction |
| 12 | Carlsbad, CA | [Targazyme](http://targazyme.com/) | Cell Therapy |
| 13 | Culver City, CA | [NantCell](http://www.nantworks.com/groups.html) | Cellular therapy |
| 14 | Emeryville, CA | [Adheren](http://www.adheren.com/) | Cancer immunotherapy |
| 15 | Fremont, CA | [Asterias Biotherapeutics (BioTime)](http://asteriasbiotherapeutics.com/) | Embryonic stem cell product |
| 16 | Irvine, CA | [Caladrius Biosciences](http://www.caladrius.com/) | Stem Cell Research Products |
| 17 | Irvine, CA | [Invitrx Therapeutics](http://www.invitrx.com/) | Autologous Stem Cell Therapy, Therapeutic & Cosmetic |
| 18 | La Jolla, CA | [Ankasa Regenerative Therapeutics](http://jlabs.jnjinnovation.com/residents/ankasa-regenerative-therapeutics) | Proprietary formulations of WNT stem cell activators |
| 19 | La Jolla, CA | [StemGenex](https://stemgenex.com/) | Stem Cell Therapy |
| 20 | La Mesa, CA | [Regen BioPharma](http://www.regenbiopharmainc.com/) | Regenerative Medicine, Cell Therapy, Small Molecule |
| 21 | Los Angeles, CA | [Iovance Biotherapeutics](http://www.iovance.com/) | Adoptive Cell Therapy |
| 22 | Menlo Park, CA | [Adicet Bio](http://www.adicetbio.com/) | Immunotherapies |
| 23 | Menlo Park, CA | [Adverum Biotechnologies](http://adverumbio.com/) | Adeno-Associated Virus Delivery, Gene Therapy |
| 24 | Mission Viejo, CA | [Generatx](http://generatx.com/) | Stem Cell Therapy |
| 25 | Mountain View, CA | [Apceth](http://www.apceth.com/) | Cell Therapies |
| 26 | Mountain View, CA | [Human Longevity Inc](http://www.humanlongevity.com/) | Genotype/Phenotype database for therapeutic/diagnostics discovery |
| 27 | Mountain View, CA | [PCT Cell Therapy (Caladrius Biosciences)](http://www.caladrius.com/) | Cell Therapy Development Support |
| 28 | Mountain View, CA | [SanBio Co Ltd](http://www.san-bio.com/) | Cellular Therapies |
| 29 | Novato, CA | [Cellular Dynamics (Fujifilm)](https://cellulardynamics.com/) | Induced Pluripotent Stem Cells |
| 30 | Palm Desert, CA | [Cell Surgical Network](http://stemcellrevolution.com/) | Stem Cell Isolation/Treatments |
| 31 | Palo Alto, CA | [Cellular Biomedicine Group](http://www.cellbiomedgroup.com/) | Cell Therapy |
| 32 | Poway, CA | [VetStem Biopharma](http://www.vet-stem.com/) | Stem Cell Therapy for Animals |
| 33 | Redwood City, CA | [Bullet Biotechnology](http://www.bulletbio.com/) | Immunotherapy |
| 34 | Redwood City, CA | [OncoMed Pharmaceuticals](http://www.oncomed.com/) | Cancer Stem Cells |
| 35 | San Carlos, CA | [BioCardia](http://www.biocardia.com/) | Personalized marrow-derived cell therapy |
| 36 | San Diego, CA | [Animal Cell Therapies](http://actcells.com/) | Veterinary Stem Cell Therapies |
| 37 | San Diego, CA | [CardioCell (Stemedica)](http://stemcardiocell.com/) | Cell Therapy |
| 38 | San Diego, CA | [Cytori Therapeutics](http://www.cytori.com/) | Cryopreservation Systems, Cellular Therapy |
| 39 | San Diego, CA | [F1 Oncology](http://f1oncology.com/) | CAR-T |
| 40 | San Diego, CA | [Histogen](http://www.histogen.com/) | Regenerative Medicine |
| 41 | San Diego, CA | [Human Longevity Inc](http://www.humanlongevity.com/) | Genotype/Phenotype database for therapeutic/diagnostics discovery |
| 42 | San Diego, CA | [Neurogeneration](https://neurogeneration.com/) | Autologous therapy |
| 43 | San Diego, CA | [Stemedica Cell Technologies](http://www.stemedica.com/) | Stem Cell Production |
| 44 | San Diego, CA | [ViaCyte](http://www.viacyte.com/) | Stem Cell Therapies |
| 45 | San Diego, CA | [Vital Therapies](http://vitaltherapies.com/) | Extracorporeal Cellular Therapy for Liver Disease |
| 46 | San Diego, CA | [Xcelthera](http://www.xcelthera.com/) | Stem Cell Therapy |
| 47 | San Francisco, CA | [Tempo Bioscience](http://www.tempobioscience.com/) | Biosensor assays, stem cells, services |
| 48 | San Mateo, CA | [Medeor Therapeutics](https://www.medeortherapeutics.com/) | Cellular Immunotherapy |
| 49 | South SF, CA | [Chimera Bioengineering](http://www.chimera.bio/) | CAR-T therapies |
| 50 | South SF, CA | [Neurona Therapeutics](http://www.neuronatherapeutics.com/) | Neuronal stem-cell therapies |
| 51 | South SF, CA | [VistaGen Therapeutics](http://www.vistagen.com/) | Stem Cell Technology |
| 52 | Yorba Linda, CA | [DaVinci Biosciences](http://www.dvbiosciences.com/) | Cellular Therapies |
| 53 | Aurora, CO | [MBC Pharma](http://www.mbcpharma.com/) | Bone-seeking Treatments |
| 54 | Aurora, CO | [Taiga Biotechnologies](http://taigabiotech.com/) | Small molecules, cell therapies |
| 55 | Aurora, CO | [Taiga Biotechnologies](http://taigabiotech.com/) | Small molecules, cell therapies |
| 56 | Alachua, FL | [AxoGen](http://www.axogeninc.com/) | Nerve Tissue Processing |
| 57 | Jupiter, FL | [BioRestorative Therapies](http://www.biorestorative.com/) | Autologous Stem Cell Therapy |
| 58 | Orlando, FL | [Immune Therapeutics](https://www.immunetherapeutics.com/) | Immunotherapy |
| 59 | Sunrise, FL | [U.S. Stem Cell](http://us-stemcell.com/en/home-2/) | Cell Therapies & Delivery |
| 60 | Tampa, FL | [Morphogenesis](http://www.morphogenesis-inc.com/) | Cells as Diagnostics |
| 61 | Tampa, FL | [Saneron CCEL Therapeutics (Cryo-Cell International, Inc)](http://www.saneron-ccel.com/) | Cellular Therapy |
| 62 | West Palm Beach, FL | [F1 Oncology](http://f1oncology.com/) | CAR-T |
| 63 | Athens, GA | [ViaCyte](http://www.viacyte.com/) | Stem Cell Therapies |
| 64 | Atlanta, GA | [Metaclipse Therapeutics](http://www.metaclipsetherapeutics.com/) | Personalized cancer therapy |
| 65 | Atlanta, GA | [SpherIngenics](http://www.spheringenics.com/) | Cellular Therapies |
| 66 | Honolulu, HI | [Tissue Genesis](http://www.tissuegenesis.com/) | Adipose Cell Isolation |
| 67 | Ames, IA | [NewLink Genetics](http://www.linkp.com/) | Cell Therapy, Small Molecules |
| 68 | Indianapolis, IN | [Cook General BioTechnology (Cook)](http://www.cookgbt.com/) | Tissue Processing, Cellular Therapy & Contract Research |
| 69 | Baton Rouge, LA | [NuPotential](http://www.nupotentialinc.com/) | Cell Line Production |
| 70 | Boston, MA | [Anagenesis Biotechnologies](http://anagenesis-biotech.com/) | Cell therapy, small molecules |
| 71 | Boston, MA | [Asymmetrex](http://asymmetrex.com/) | Adult stem cell 'production' |
| 72 | Boston, MA | [Celyad](http://www.celyad.com/) | Stem Cell Differentiation |
| 73 | Boston, MA | [Inform Genomics](http://www.informgenomics.com/) | Genomic-based medicines |
| 74 | Boston, MA | [ORIG3N](http://www.orig3n.com/) | iPS cell therapy |
| 75 | Boston, MA | [Vor BioPharma](http://www.vorbiopharma.com/) | CAR-T Stem Cell Therapy |
| 76 | Boston, MA | [Ziopharm Oncology](http://www.ziopharm.com/) | Cellcular Therapies |
| 77 | Cambridge, MA | [AVROBIO](http://www.avrobio.com/) | Cellular & Gene Therapies |
| 78 | Cambridge, MA | [eGenesis](http://www.egenesisbio.com/) | CRISPR for xenotransplantation |
| 79 | Cambridge, MA | [Magenta Therapeutics](https://www.magentatx.com/) | Stem Cell, Bone Marrow Transplant Technology |
| 80 | Cambridge, MA | [MirImmune (Rxi Pharma)](http://mirimmunebio.com/wp1/) | Cancer Immunotherapy |
| 81 | Cambridge, MA | [Neon Therapeutics](http://neontherapeutics.com/) | Vaccines & T cell therapies |
| 82 | Cambridge, MA | [OvaScience](http://www.ovascience.com/) | Fertility, Mitochondria Transplantation |
| 83 | Cambridge, MA | [Pathfinder Cell Therapy](http://www.pathfindercelltherapy.com/) | Cell-based Therapy |
| 84 | Cambridge, MA | [Rubius Therapeutics](http://www.rubiustx.com/) | Enucleated cell therapeutics |
| 85 | Cambridge, MA | [Semma Therapeutics](http://www.semma-tx.com/) | Diabetes Stem Cell Treatments |
| 86 | Cambridge, MA | [TCR2 Therapeutics](http://www.tcr2.com/) | T Cell Immunotherapy |
| 87 | Cambridge, MA | [Unum Therapeutics](http://www.unumrx.com/) | cellular immunotherapies |
| 88 | Cambridge, MA | [Vericel](http://vcel.com/) | Cellular Therapy |
| 89 | Natick, MA | [RegenoCELL Therapeutics](http://www.regenocell.com/) | Stem Cell Therapy |
| 90 | Waltham, MA | [Juno Therapeutics](https://www.junotherapeutics.com/) | Immunotherapy, CAR-T |
| 91 | Waltham, MA | [Neovii Biotech](http://neovii.com/) | Stem Cell Therapy, Biologics |
| 92 | Waltham, MA | [ViaCord (PerkinElmer)](http://www.viacord.com/) | Cord Blood Banking |
| 93 | Bethesda, MD | [AnGes](http://www.anges-mg.com/en/) | Gene therapy |
| 94 | Clarksville, MD | [Neuronascent](http://www.neuronascent.com/) | Small molecules, stem cells |
| 95 | Columbia, MD | [Osiris Therapeutics](http://www.osiristx.com/) | Cellular Matrix Treatments |
| 96 | Gaithersburg, MD | [NexImmune](http://www.neximmune.com/) | Artificially engineered antigen-presenting cells |
| 97 | Gaithersburg, MD | [Trevigen](http://www.trevigen.com/) | Cell Assays |
| 98 | Germantown, MD | [Neuralstem](http://www.neuralstem.com/) | Stem Cell Technology |
| 99 | Germantown, MD | [Orgenesis](http://www.orgenesis.com/) | Autologous Cellular Conversion |
| 100 | Rockville, MD | [TissueGene](http://www.tissuegene.com/) | Regenerative therapies for orthopedic disorders |
| 101 | Ann Arbor, MI | [Vericel](http://vcel.com/) | Cellular Products |
| 102 | Research Triangle Park, NC | [ZenBio](http://www.zen-bio.com/) | Human Cells, Media, Kits, Tissues |
| 103 | Winston-Salem, NC | [Cellf BIO](http://www.cellfbio.com/) | Regenerative medicine-based therapy for fecal incontinence |
| 104 | Winston-Salem, NC | [Plureon](http://www.plureon.com/) | Pluripotent stem cells derived from amniotic fluid and placenta |
| 105 | Lebanon, NH | [Celdara Medical](http://www.celdaramedical.com/) | Cellular therapy, diagnostics |
| 106 | Allendale, NJ | [PCT Cell Therapy (Caladrius Biosciences)](http://www.caladrius.com/) | Cell Therapy Development Support |
| 107 | Basking Ridge, NJ | [Caladrius Biosciences](http://www.caladrius.com/) | Adult Stem Cell Storage |
| 108 | Eatontown, NJ | [Personal Cell Sciences](http://personalcellsciences.com/) | Autologous stem cell firming serum for aesthetics |
| 109 | Hackensack, NJ | [BrainStorm Cell Therapeutics](http://www.brainstorm-cell.com/) | autologous, adult stem cell therapy technology |
| 110 | Little Falls, NJ | [Regenicin](http://www.regenicin.com/) | Autologous skin cell therapy |
| 111 | New Brunswick, NJ | [Celvive](http://www.celvive.com/) | Cell Therapy |
| 112 | New Brunswick, NJ | [ChromoCell](http://www.chromocell.com/) | Cell Therapy |
| 113 | North Brunswick | [ChromoCell](http://www.chromocell.com/) | Drug Discovery, Cellular Therapy |
| 114 | New York, NY | [BlueRock Therapeutics](http://bluerocktx.com/) | Stem Cell Therapies |
| 115 | New York, NY | [Caladrius Biosciences](http://www.caladrius.com/) | Adult Stem Cell Storage |
| 116 | New York, NY | [DanDrit Biotechnology](http://www.dandrit.com/) | Cancer Vaccines |
| 117 | New York, NY | [Dompé](http://www.dompe.com/en/) | Biologics, Small Molecules, OTC |
| 118 | New York, NY | [Helocyte (Fortress Bioecth)](http://www.helocyte.com/) | Cytomegalovirus, oncology immunotherapies |
| 119 | New York , NY | [Mesoblast](http://www.mesoblast.com/) | Regenerative Medicine |
| 120 | New York, NY | [Stemline Therapeutics](http://www.stemline.com/) | Cancer Stem Cells |
| 121 | Exton, PA | [Fibrocell Science](http://fibrocell.com/) | Personalized Skin Therapy |
| 122 | Philadelphia, PA | [Adaptimmune Therapeutics](http://www.adaptimmune.com/) | T Cell Cancer Therapy |
| 123 | Pittsburgh, PA | [Celsense](http://celsense.com/) | Real-time MRI detection of inflammation and cellular therapeutics |
| 124 | Providence, RI | [Semma Therapeutics](http://www.semma-tx.com/) | Diabetes Stem Cell Treatments |
| 125 | Austin, TX | [TeVideo Biodevices](http://tevidobiodevices.com/) | Cell therapy for breast reconstruction |
| 126 | Floresville, TX | [Targazyme](http://targazyme.com/) | Cell Therapy |
| 127 | Houston, TX | [Bellicum Pharmaceuticals](http://www.bellicum.com/) | Cellular Therapy |
| 128 | Houston, TX | [Inform Genomics](http://www.informgenomics.com/) | Genomic-based medicines |
| 129 | Houston, TX | [InGeneron](http://ingeneron.com/) | Stem cell collection and processing technology |
| 130 | San Antonio, TX | [Cytori Therapeutics](http://www.cytori.com/) | Cryopreservation Systems, Cellular Therapy |
| 131 | San Antonio, TX | [StemBioSys](http://www.stembiosys.com/) | Stem Cells |
| 132 | The Woodlands, TX | [Opexa Therapeutics](http://www.opexatherapeutics.com/) | Cell Therapy |
| 133 | Salt Lake City, UT | [DiscGenics](http://discgenics.com/Home.html) | Spinal Stem Cell Therapies |
| 134 | Redmond, WA | [Astarte Biologics](http://www.astartebio.com/) | Cell Products |
| 135 | Seattle, WA | [AVM Biotechnology](http://avmbiotech.com/) | Stem Cell Technologies |
| 136 | Seattle, WA | [Immusoft](http://immusoft.com/) | Autologous B cell-conditioning |
| 137 | Seattle, WA | [Juno Therapeutics](https://www.junotherapeutics.com/) | Immunotherapy, CAR-T |
| 138 | Seattle, WA | [Nohla Therapeutics](http://nohlatherapeutics.com/) | Universal donor cell therapy |
| 139 | Seattle, WA | [Universal Cells](http://www.universalcells.com/) | Non-immunogenic cells |
| 140 | Madison, WI | [Cell Line Genetics](http://www.clgenetics.com/) | Pharmaceutical Services |
| 141 | Madison, WI | [Cellular Dynamics (Fujifilm)](http://www.cellulardynamics.com/) | Induced Pluripotent Stem Cells |
| 142 | Madison, WI | [Stratatech (Mallinckrodt)](http://www.stratatechcorp.com/) | Cell Therapy, Tissue Engineering |
|  | **Canada** |  |  |
| 143 | Vancouver, BC | [RepliCel Life Sciences](http://www.replicel.com/) | Autologous cell therapies for healing |
| 144 | Toronto, ON | [AVROBIO](http://www.avrobio.com/) | Cellular & Gene Therapies |
| 145 | Toronto, ON | [ExCellThera](http://excellthera.ca/) | Growing blood stem cells for therapeutic use |
| 146 | Toronto, ON | [Trillium Therapeutics](http://trilliumtherapeutics.com/trillium-home/default.aspx) | Stem Cell Therapy |
| 147 | Vancouver | Stem Cell Technologies | iPSCs research products |
|  | **European Union** |  |  |
| 148 | Gosselies, Belgium | [Bone Therapeutics](http://www.bonetherapeutics.com/en) | Cell Therapy |
| 149 | Mont-Saint-Guibert, Belgium | [Celyad](http://www.celyad.com/) | Stem Cell Differentiation |
| 150 | Mont-Saint-Guibert, Belgium | [Promethera Biosciences](http://www.promethera.com/) | Liver disease treatments |
| 151 | Copenhagen, Denmark | [DanDrit Biotechnology](http://www.dandrit.com/) | Cancer Vaccines |
| 152 | Corbeil-Essonnes, France | [I-stem](http://www.istem.eu/en/) | Human pluripotent stem cells, of embryonic origin or iPSCs |
| 153 | Fontenay-aux-Roses, France | [Brainvectis](http://www.brainvectis.com/) | Viral Vector Therapy |
| 154 | Illkirch Graffenstaden, France | [Anagenesis Biotechnologies](http://anagenesis-biotech.com/) | Cell therapy, small molecules |
| 155 | Mulhouse, France | [CellProthera](http://www.cellprothera.com/) | Stem cell transplant for cardiac conditions |
| 156 | Mulhouse, France | [CellProthera](http://www.cellprothera.com/) | Stem cell transplant for cardiac conditions |
| 157 | Toulouse, France | [InvivoGen Therapeutics](http://www.invivogen-therapeutics.com/) | Gene and Immunotherapies |
| 158 | Valbonne , France | [TxCell](http://www.txcell.com/index.php/en/) | Personalized cellular immunotherapies |
| 159 | Cologne, Germany | [Axiogenesis](http://www.axiogenesis.com/) | Stem Cell Products |
| 160 | Graefelfing, Germany | [Neovii Biotech](http://neovii.com/) | Stem Cell Therapy, Biologics |
| 161 | Munich, Germany | [Apceth](http://www.apceth.com/) | Cell Therapies |
| 162 | Ottobrunn, Germany | [Apceth](http://www.apceth.com/) | Cell Therapies |
| 163 | Tuebingen, Germany | [Immatics Biotechnologies](http://immatics.com/) | Cancer immunotherapy |
| 164 | L'Aquila, Italy | [Dompé](http://www.dompe.com/en/) | Biologics, Small Molecules, OTC |
| 165 | Milan, Italy | [Molecular Medicine](http://www.molmed.com/) | Biologics, Small Molecules, Cellular Therapy |
| 166 | Milano, Italy | [Dompé](http://www.dompe.com/en/) | Biologics, Small Molecules, OTC |
| 167 | Napoli, Italy | [Dompé](http://www.dompe.com/en/) | Biologics, Small Molecules, OTC |
| 168 | Rome, Italy | [Okairos](http://www.okairos.com/e/index.php) | T-Cell Based Vaccines |
| 169 | Vecchiano, Italy | [Galileo Research](http://www.galileoresearch.it/) | Cell Therapy, Contract Research |
| 170 | Amsterdam, Netherlands | [Kiadis Pharma](http://www.kiadis.com/) | Stem Cell Treatment |
| 171 | Maastricht, Netherlands | [CiMaas](http://cimaas.com/) | Cellular Immunotherapy |
| 172 | Maastricht, Netherlands | [Pharma Cell](http://pharmacell.nl/) | Contract Cellular Manufacturing |
| 173 | Lodz, Poland | [Celther Polska](http://www.celther.pl/) | Stem Cell Lines |
| 174 | Viken, Sweden | [Tikomed](http://tikomed.com/) | Regenerative Medicine |
| 175 | Basel, Switzerland | [Targazyme](http://targazyme.com/) | Cell Therapy |
| 176 | Rapperswil, Switzerland | [Neovii Biotech](http://neovii.com/) | Stem Cell Therapy, Biologics |
| 177 | Abingdon, UK | [Adaptimmune Therapeutics](http://www.adaptimmune.com/) | T Cell Cancer Therapy |
| 178 | Bridgend, UK | [ReNeuron](http://www.reneuron.com/) | Stem Cell Therapies |
| 179 | Cambridge, UK | [OxStem](http://www.oxstem.co.uk/) | Stem Cell Drugs |
| 180 | Cambridge, UK | [Talisman Therapeutics](http://www.talisman-therapeutics.com/) | Alzheimer's Stem Cell Model |
| 181 | Cardiff, UK | [Celixir](http://www.celixir.com/) | Regenerative Medicine |
| 182 | Croydon, UK | [AnGes](http://www.anges-mg.com/en/) | Gene therapy |
| 183 | Deeside, UK | [Cytori Therapeutics](http://www.cytori.com/) | Cryopreservation Systems, Cellular Therapy |
| 184 | Edinburgh, UK | [Roslin Cells](http://www.roslincells.com/) | Stem Cells |
| 185 | London, UK | [Cell Medica](http://www.cellmedica.co.uk/) | Personalized cellular immunotherapies |
| 186 | Machester, UK | [Cellular Therapeutics](http://www.cellulartherapeutics.co.uk/) | Cell therapies |
| 187 | Manchester, UK | [Intercytex](http://www.intercytex.com/) | Cell-Based Products |
| 188 | Melbourn, UK | [Avita Medical](http://www.avitamedical.com/) | Autologous Skin Cell Treatment, Drug Delivery |
| 189 | Vienna Austria | Activartis | Dendritic cell-based cance immunotherapy |
| 190 | Vienna Austria | Aposcience | Treatments composed of mixture of cytokines, growth factors and other active components |
|  | **People’s Republic of China** |  |  |
| 191 | Admiralty, China | [Cellular Biomedicine Group](http://www.cellbiomedgroup.com/) | Cell Therapy |
| 192 | Nanjing, China | [Nanjing Legend](http://www.legendpharm.com/) | Small Molecules, CAR-T |
| 193 | Shanghai, China | [Cellular Biomedicine Group](http://www.cellbiomedgroup.com/) | Cell Therapy |
| 194 | Taipei City, Taiwan, China | [TaiwanBio](http://www.taiwan-bio-thera.com/#!taiwan-bio-therapeutics/c1l5i) | Allogeneic mesenchymal stem cell therapy |
| 195 | Beijing, China | Jieya Laifu Biotechnology | J-1 allogeneic acellular dermal matrix:  To repair oral mucosal defects, soft tissue defects. |
| 196 | Beijing, China | Jieya Laifu Biotechnology | AlloDerm: To repair defect of human derma |
| 197 | Beijing, China | Qingyuan Albert tissue engineering biological technology | Rhino (acellular dermal matrix medical tissue patch) To repair oral mucosa and soft tissue defects |
| 198 | Chongqing, China | Zongshen Junhui Biotechnology | Artificial skin – gene transfection pigskin: To repair burns and other trauma wound coverage |
| 199 | Qidong, Hunan, China | Oriental Medicine Research Institute | Acellular dermal matrix dressings: To repair superficial II degree burn wounds |
| 200 | Shaanxi, China | Eyre skin Biological Engineering | To repair deep II degree burn wound, not more than III degree burn wound 20 cm2 |
| 201 | Zhenghai, China | BiotechnologyYantai Zhenghai: Skin repair film | To repair various causes dermal wound repair defects. |
| 202 | henghai, China | BiotechnologyYantai Zhenghai: Biofilm | To repair a variety of causes dura (spinal) membrane defects |
| 203 | Zhenghai, China | BiotechnologyYantai Zhenghai: Dental film | To repair various causes shallow intraoral soft tissue defect repair |
| 204 | Guangdong, China | Biological Technology of Guangdong Grandhope | General thoracic surgical repair film |
| 205 | Guangdong, China | Biological Technology of Guangdong Grandhope: Sterile biological care record film | Skin burn, burn and trauma, skin defects |
| 206 | Guangdong, China | Biological Technology of Guangdong Grandhope: Biotypes dura (spinal) membrane patch | To repair hard brain (spinal) membrane defect |
| 207 | Beijing, China | Datsing Bio-Tech: Allogeneic bone grafts | Dental implants, to repair broken bones and bone that has not yet healed |
|  | **India** |  |  |
| 208 | Bangalore, KA | [Stempeutics](http://www.stempeutics.com/) | Stem cell based products |
| 209 | Delhi | [StemGenn Therapeutics](http://www.stemgenn.com/) | Stem Cell Products/Therapies |
| 210 | Gurgaon | [APAC Biotech](http://www.apacbiotech.com/) | Immunocellular Therapy |
| 211 | Noida, UP | [Advancells](http://www.advancells.com/) | Stem Cell Treatments |
| 212 | South Delhi | Nutech Mediworld | HESCs Therapy |
| 213 | Delhi | Reliance Life Sciences | Stem Cell Therapies |
| 214 | Delhi | Life Cell | Stem Cell Therapies |
| 215 | Delhi | Cryobanks India | Stem Cell Therapies |
| 216 | Delhi | Trans-Scell Biologics | Stem Cell Therapies |
|  | **Japan** |  |  |
| 217 | Ibaraki | [DNAVEC](http://www.dnavec.co.jp/jp/) | Viral Vectors & Gene Therapy |
| 218 | Osaka | [AnGes](http://www.anges-mg.com/en/) | Gene therapy |
| 219 | Tokyo | [AnGes](http://www.anges-mg.com/en/) | Gene therapy |
| 220 | Tokyo | [Cytori Therapeutics](http://www.cytori.com/) | Cryopreservation Systems, Cellular Therapy |
| 221 | Kyoto | Megakaryon | Mass-production of platelets from iPS cells |
| 222 | Kyoto | [ReproCELL](https://www.reprocell.com/en) | Commercial iPSC products; human iPSC-derived cardiomyocytes |
|  | **Israel** |  |  |
| 223 | Jerusalem | [Cell Cure Neurosciences (BioTime)](http://www.cellcureneurosciences.com/) | Cell Therapy |
| 224 | Jerusalem | [Gamida Cell](http://www.gamida-cell.com/) | Stem Cell Therapy |
| 225 | Kfar Saba | [Cellect Biosciences](http://cellect.co/) | Regenerative Medicine |
| 226 | Kiryat Aryeh, | BrainStorm Cell | Autologous, adult stem cell therapy |
| 227 | Israel | Therapeutics | Technology |
| 228 | Ness Ziona | [Kadimastem](http://www.kadimastem.com/) | Stem cell therapeutics |
|  | **South Korea** |  |  |
| 229 | Seoul | [Eutilex](http://eutilex.com/eng/home.php) | T cell and antibody therapies |
| 230 | Seoul | [MEDIPOST](http://www.medi-post.com/) | Stem cell technology & regenerative medicine |
|  | **Singapore** |  |  |
| 231 | Singapore | [Human Longevity Inc](http://www.humanlongevity.com/) | Genotype/Phenotype database for therapeutic/diagnostics discovery |
| 232 | Singapore | [Cell Research Corp](http://www.cellresearchcorp.com/) | Cord Blood Stem Cells & products |
|  | **New Zealand** |  |  |
| 233 | Auckland | [Living Cell Technologies](http://www.lctglobal.com/) | Regenerative Medicine |
|  | **Brazil** |  |  |
| 234 | São Paulo | [PluriCell](http://www.pluricellbiotech.com.br/en/home-en/) | iPS Cells |
|  | **Malaysia** |  |  |
| 235 | Cyberjaya | CryoCord Sdn Bhd | Cord blood and stem cell banking |
| 236 | Kuala Lumpur | StemLife Berhad | Cord blood and stem cell banking |
| 237 | Kuala Lumpur | Stempeutics Research Sdn. Bhd. | Therapeutics and theraphy in the field of regenerative medicine |
| 238 | Kuala Lumpur | CellSafe International Sdn. Bhd. | [Cord Blood Stem Cell Cryogenic Preservation Services](https://www.google.com.pg/url?sa=t&rct=j&q=&esrc=s&source=web&cd=1&cad=rja&uact=8&ved=0ahUKEwiE6fbh8ZvWAhXHso8KHXRkDvoQ_UUIAjAA&url=http%3A%2F%2Fcellsafegroup.com%2F&usg=AFQjCNFKDNOZdhnhQb2zgJH2Fgnm3DG3tw) |
| 239 | Kuala Lumpur | Stemtech Sdn Bhd | The Stem Cell Nutrition |
| 240 | Kuala Lumpur | Cell Tissue Technology Sdn Bhd | Tissue engineered and cell based products |
